# The Protein *O*-glucosyltransferase Rumi Modifies Eyes Shut to Promote Rhabdomere Separation in *Drosophila*

**DOI:** 10.1371/journal.pgen.1004795

**Published:** 2014-11-20

**Authors:** Amanda R. Haltom, Tom V. Lee, Beth M. Harvey, Jessica Leonardi, Yi-Jiun Chen, Yang Hong, Robert S. Haltiwanger, Hamed Jafar-Nejad

**Affiliations:** 1 Program in Genes & Development, The University of Texas Graduate School of Biomedical Sciences, Houston, Texas, United States of America; 2 Department of Molecular & Human Genetics, Baylor College of Medicine, Houston, Texas, United States of America; 3 Department of Biochemistry and Cell Biology, Stony Brook University, Stony Brook, New York, United States of America; 4 Program in Developmental Biology, Baylor College of Medicine, Houston, Texas, United States of America; 5 Department of Cell Biology, University of Pittsburgh School of Medicine, Pittsburgh, Pennsylvania, United States of America; Harvard Medical School, Howard Hughes Medical Institute, United States of America

## Abstract

The protein *O*-glucosyltransferase Rumi/POGLUT1 regulates *Drosophila* Notch signaling by adding *O*-glucose residues to the Notch extracellular domain. Rumi has other predicted targets including Crumbs (Crb) and Eyes shut (Eys), both of which are involved in photoreceptor development. However, whether Rumi is required for the function of Crb and Eys remains unknown. Here we report that in the absence of Rumi or its enzymatic activity, several rhabdomeres in each ommatidium fail to separate from one another in a Notch-independent manner. Mass spectral analysis indicates the presence of *O*-glucose on Crb and Eys. However, mutating all *O*-glucosylation sites in a *crb* knock-in allele does not cause rhabdomere attachment, ruling out Crb as a biologically-relevant Rumi target in this process. In contrast, *eys* and *rumi* exhibit a dosage-sensitive genetic interaction. In addition, although in wild-type ommatidia most of the Eys protein is found in the inter-rhabdomeral space (IRS), in *rumi* mutants a significant fraction of Eys remains in the photoreceptor cells. The intracellular accumulation of Eys and the IRS defect worsen in *rumi* mutants raised at a higher temperature, and are accompanied by a ∼50% decrease in the total level of Eys. Moreover, removing one copy of an endoplasmic reticulum chaperone enhances the rhabdomere attachment in *rumi* mutant animals. Altogether, our data suggest that *O*-glucosylation of Eys by Rumi ensures rhabdomere separation by promoting proper Eys folding and stability in a critical time window during the mid-pupal stage. Human EYS, which is mutated in patients with autosomal recessive retinitis pigmentosa, also harbors multiple Rumi target sites. Therefore, the role of *O*-glucose in regulating Eys may be conserved.

## Author Summary

Glycosylation (addition of sugars to proteins and other organic molecules) is important for protein function and animal development. Each form of glycosylation is usually present on multiple proteins. Therefore, a major challenge in understanding the role of sugars in animal development is to identify which protein(s) modified by a specific sugar require the sugar modification for proper functionality. We have previously shown that an enzyme called Rumi adds glucose molecules to an important cell surface receptor called Notch, and that glucose plays a key role in the function of Notch both in fruit flies and in mammals. Using fruit flies, we have now identified a new Rumi target called “Eyes shut”, a secreted protein with a critical role in the optical isolation of neighboring photoreceptors in the fly eye. Our data suggest that glucose molecules on Eyes shut promote its folding and stability in a critical time window during eye development. Mutations in human Eyes shut result in a devastating form of retinal degeneration and loss of vision. Since human Eyes shut is also predicted to harbor glucose molecules, our work provides a framework to explore the role of sugar modifications in the biology of a human disease protein.

## Introduction

Diurnal insects possess “apposition eyes” in which ommatidia are optically isolated from each other [1], [2]. In most diurnal insects like honeybee and butterflies, the apical rhodopsin-housing structures of each ommatidium—the rhabdomeres—are fused at the center. This allows the group of photoreceptors in each ommatidium to act as a single optical device [1]. A modification of the apposition eye arose during insect evolution in dipteran flies, where an extracellular lumen called the inter-rhabdomeral space (IRS) forms to separate and optically isolate the rhabdomeres in each ommatidium from one another. Due to this structural modification and the accompanying regrouping of photoreceptor axons among neighboring ommatidia, information from photoreceptor cells that receive light from the same point in the space merge on the same postsynaptic targets in the lamina [3]. This type of eye is referred to as a neural superposition eye, and these improvements allow for increased light sensitivity without sacrificing resolution [1], [4].

Separation of the rhabdomeres in flies requires an evolutionarily conserved secreted glycoprotein called Eyes shut (Eys; also called Spacemaker). *eys* mutant flies lack the IRS and exhibit an altered photoreceptor organization that resembles the closed rhabdom of other insects like honeybees and mosquitos [5], [6]. Eys is secreted from the stalk membrane of the photoreceptor cells in an Ire1-dependent but Sec6-independent manner to separate the rhabdomeres and open the IRS [5], [7]. *Drosophila eys* functions together with three other genes, *crumbs* (*crb*), *prominin* (*prom*) and *chaoptin* (*chp*), to regulate rhabdomere separation and IRS size [5], [6], [8], [9]. Genetic experiments have established that *prom* and *eys* promote rhabdomere separation but *chp* and *crb* promote rhabdomere adhesion, and that the balance between their activities results in proper IRS formation [6], [8], [9].

The Crb extracellular domain and the Eys protein are primarily composed of epidermal growth factor-like (EGF) repeats and Laminin G domains [5], [6], [10], [11]. However, the role of these protein domains and their posttranslational modifications in the function Eys and Crb is unknown. Five of the Eys EGF repeats and seven of the Crb EGF repeats contain the C^1^XSX(P/A)C^2^ consensus sequence, which predicts the addition of an *O*-linked glucose by the protein *O*-glucosyltransferase Rumi (POGLUT1 in mammals) [12], [13]. Mutations in *rumi* were first isolated in a genetic screen for regulators of sensory organ development in *Drosophila* [12]. When raised at 18°C, *rumi* mutants are viable and only show a mild loss of Notch signaling in certain contexts including bristle lateral inhibition and leg joint formation [12], [14]. However, when raised at higher temperatures the mutant animals show a broad and severe loss of Notch signaling, until 28–30°C, at which loss of *rumi* becomes larval lethal [12], [14], [15]. Mice lacking the Rumi homolog POGLUT1 die at early embryonic stages (at or before E9.5) and some of the defects observed in mutant embryos are characteristic of loss of Notch signaling [16]. Moreover, transgenic expression of human POGLUT1 in flies rescues the *rumi* null phenotypes, indicating that the function of Rumi is conserved [17]. *Drosophila* Notch has 18 Rumi target sites in its extracellular domain, most of which have been confirmed to harbor *O*-glucose residues [12], [18]. Moreover, serine-to-alanine mutations in the Rumi target sites of Notch result in a temperature-sensitive loss of Notch signaling [14], establishing Notch as a biologically-relevant target of Rumi in flies. However, whether Rumi and its glucosyltransferase activity are required for the function of its other potential targets like Eys and Crb and for rhabdomere separation remained unknown.

Here, we present evidence indicating that the enzymatic activity of Rumi is required for the separation of rhabdomeres in the *Drosophila* eye. When raised at 18°C, animals homozygous for a null allele of *rumi* or for a missense mutation that abolishes its protein *O*-glucosyltransferase activity show a highly penetrant rhabdomere attachment phenotype that cannot be explained by loss of *O*-glucose from Notch. Mass spectral analysis indicates that both Crb and Eys harbor *O*-glucose when expressed in a fly cell line. However, genetic experiments rule out Crb as a target of Rumi during rhabdomere separation. Our data indicate that *O*-glucosylation of Eys by Rumi promotes Eys folding and stability and thereby ensures that enough Eys is secreted into the IRS in a critical time window during the mid-pupal stage to fully separate the rhabdomeres.

## Results

### Mutations in *rumi* result in a rhabdomere attachment phenotype which starts in the mid-pupal stage

**Figure 1:**
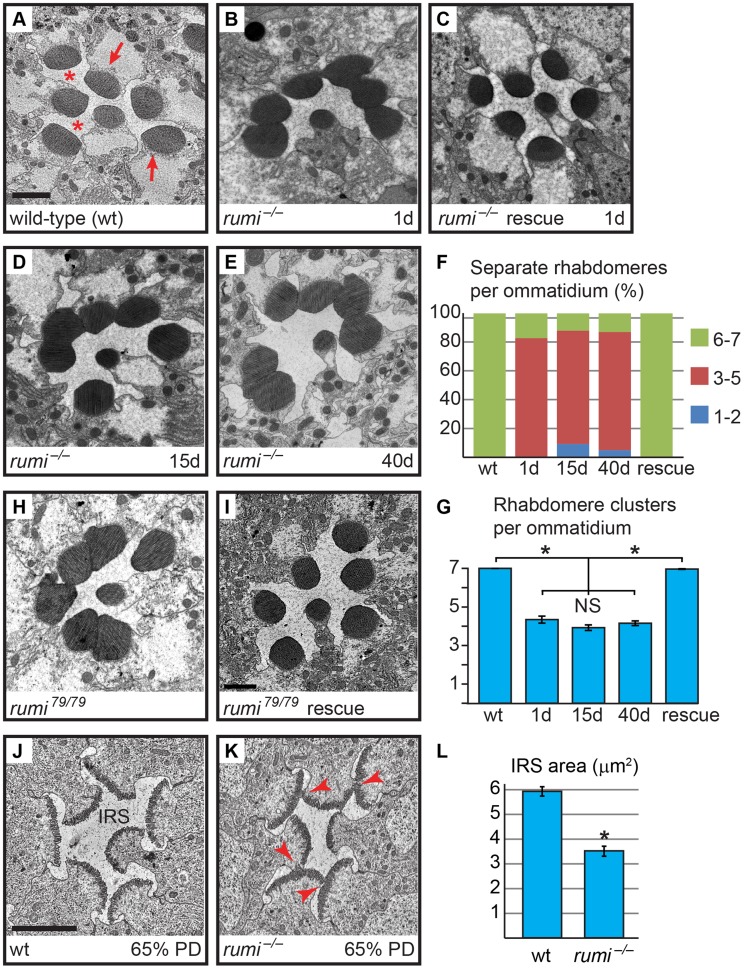
Loss of the enzymatic function of Rumi results in rhabdomere adhesion. Shown are electron micrographs of a single ommatidium from adult (A–E,H,I) or 65% PD (J,K) from the indicated genotypes. All animals were raised at 18°C. (A) Wild-type. Arrows indicate rhabdomeres and asterisks indicate the IRS. Scale bar: 2 µm. (B) 1-day old *rumi^−/−^*. Note the attachment in the neighboring rhabdomeres. (C) 1-day old *rumi^−/−^* expressing a wild-type *P{rumi^gt-FLAG^}* genomic transgene. (D,E) The *rumi^−/−^* rhabdomere attachment phenotype does not change with age, since 15-day old (D) and 40-day old (E) animals show a similar degree of attachment. (F) Percentage of number of individual rhabdomeres per ommatidia for various genotypes. At least three animals were used for each genotype. The number of ommatidia examined for each genotype is as follows: wt (50), 1d (35), 15d (66), 40d (85), rescue (126). (G) Quantification of average individual rhabdomere number per ommatidium for the data shown in F. Rhabdomere attachments in 1-day, 15-day and 40-day old *rumi* animals are not significantly different from one another, but are significantly different from wild-type and rescued animals (*P<0.0001). NS, not significant. (H,I) The enzymatically inactive allele *rumi^79^* also shows a rhabdomere attachment phenotype (H), which can be rescued by one copy of the wild-type *P{rumi^gt-FLAG^}* genomic transgene (I). (J,K) Ommatidia from animals at 65% PD from wild-type (J) and *rumi^−/−^* (K). Arrowheads in (K) indicate points of rhabdomere attachment. (L) Means ± SEM of IRS area in wild-type and *rumi^−/−^* at 65% PD. IRS areas were measured using ImageJ software. Unpaired *t*-test was used to compare wt (n = 14) and *rumi^−/−^* (n = 24) IRS, *P<0.0001.

When raised at 18°C, *rumi* mutant animals are viable and show only a mild loss of Notch signaling in some contexts [12], [14]. To explore whether Rumi plays a role in rhabdomere morphogenesis and IRS formation, we raised animals homozygous for the protein-null allele *rumi^Δ26^* (*rumi^−^*) in ambient light at 18°C and performed transmission electron microscopy (TEM) on adult fly eyes. In cross sections of wild-type retinas, the rhabdomeres of the seven visible photoreceptor cells are separated from neighboring rhabdomeres by the IRS [19] (Figure 1A and 1F). However, 1-day old *rumi^−/−^* animals exhibited a moderate, yet 100% penetrant, rhabdomere attachment phenotype, i.e. attachment of two or more rhabdomeres per ommatidium (Figure 1B and 1F). This phenotype can be fully rescued by *P{rumi^gt-FLAG^}* (Figure 1C and 1F), a genomic transgene expressing a FLAG-tagged version of Rumi [12], indicating that attachment of rhabdomeres observed in *rumi^−/−^* flies is due to the loss of *rumi*. Sections of *rumi^−/−^* animals at 15 and 40 days of age show a similar degree of rhabdomere attachment, suggesting that the phenotype is not age-dependent (Figure 1D–G). Together, these observations indicate that Rumi is required for optical isolation of individual photoreceptors in the *Drosophila* eye.

We have previously shown that Rumi primarily regulates Notch signaling through its protein *O*-glucosyltransferase activity [12], [14]. We wondered whether the enzymatic activity of Rumi is also required for rhabdomere separation. To test this, we performed TEM on adult *Drosophila* homozygous for *rumi^79^*, a severe hypomorphic allele harboring a missense mutation which abolishes the enzymatic activity of Rumi but does not affect its expression level or stability [12], [17]. *rumi^79/79^* animals raised at 18°C also exhibit rhabdomere attachment in all ommatidia examined (Figure 1H; n>50). Surprisingly, the average number of separate rhabdomeres per ommatidium was somewhat lower in *rumi^79/79^* animals (3.41±0.15) compared to *rumi^−/−^* animals (4.11±0.08), indicating that the rhabdomere attachment phenotype is slightly more severe in *rumi^79/79^* animals compared to *rumi^−/−^* animals. Statistical analysis indicated that the difference between *rumi^79/79^* and *rumi^−/−^* average rhabdomere number per ommatidia is significant (*P*<0.0001). Given that *rumi^Δ26^* is a protein-null allele [12], these data suggest that *rumi^79^* might have a dominant negative effect in the context of rhabdomere separation. However, one copy of the *P{rumi^gt-FLAG^}* genomic transgene was able to fully rescue the rhabdomere attachment phenotype of *rumi^79/79^* animals (Figure 1I, n>50). Moreover, overexpression of Rumi-G189E, which is the protein product of *rumi^79^* [12], did not result in any rhabdomere separation defects, similar to overexpression of wild-type Rumi (Figure S1). Together, these observations suggest that *rumi^79^* is not likely to be a dominant negative allele. Since *rumi^79^* was generated in an EMS screen but *rumi^Δ26^* is the product of *P*-element excision, the modest worsening of the rhabdomere attachment in *rumi^79^* might be due to a genetic background effect. Taken together, these observations indicate that enzymatic activity of Rumi is required for the separation of rhabdomeres in the fly eye.

Rhabdomere morphogenesis and IRS formation occur during the second half of pupal development [19]. Until 37% pupal development (PD), the apical surfaces of photoreceptors are attached to one another and do not exhibit any microvillar structures [19]. Around 55% PD, short microvilli and neighboring stalk membranes can be seen at the apical surfaces of the developing photoreceptors, and a thin IRS has formed [19]. By 65% PD, the rhabdomeres are clearly separated from one another by the IRS (Figure 1J). Because one-day old adult *rumi* retinas have a well-formed IRS but exhibit rhabdomere attachment (Figure 1B), we asked whether the absence of Rumi prevents rhabdomere separation during pupal development, or whether they initially separate but subsequently attach as the pupal eye assumes its adult structure. To address this question, we performed TEM on 65% PD *rumi^−/−^* retinas grown at 18°C, and found that by 65% PD, each *rumi* photoreceptor harbors distinct stalk membrane and rhabdomere structures (Figure 1K). The IRS has formed but the average IRS size in mutant ommatidia (3.52±0.20 µm^2^) is 59% of the average IRS size in wild-type ommatidia (5.95±0.19 µm^2^) at a similar stage raised at the same temperature (Figure 1L, *P*<0.0001). Although the apical surfaces of photoreceptors adjacent to the IRS appear separated from one another, multiple local adhesions persist between the microvillar membranes of neighboring rhabdomeres (and occasionally opposing rhabdomeres) in each ommatidium (Figure 1K, arrowheads). These observations indicate that the *rumi* rhabdomere attachment phenotypes are evident early during rhabdomere morphogenesis and strongly suggest that proper rhabdomere separation never occurs in *rumi^−/−^* animals.

### Loss of *O*-glucose from Notch cannot explain the rhabdomere attachment phenotype of *rumi* animals

**Figure 2:**
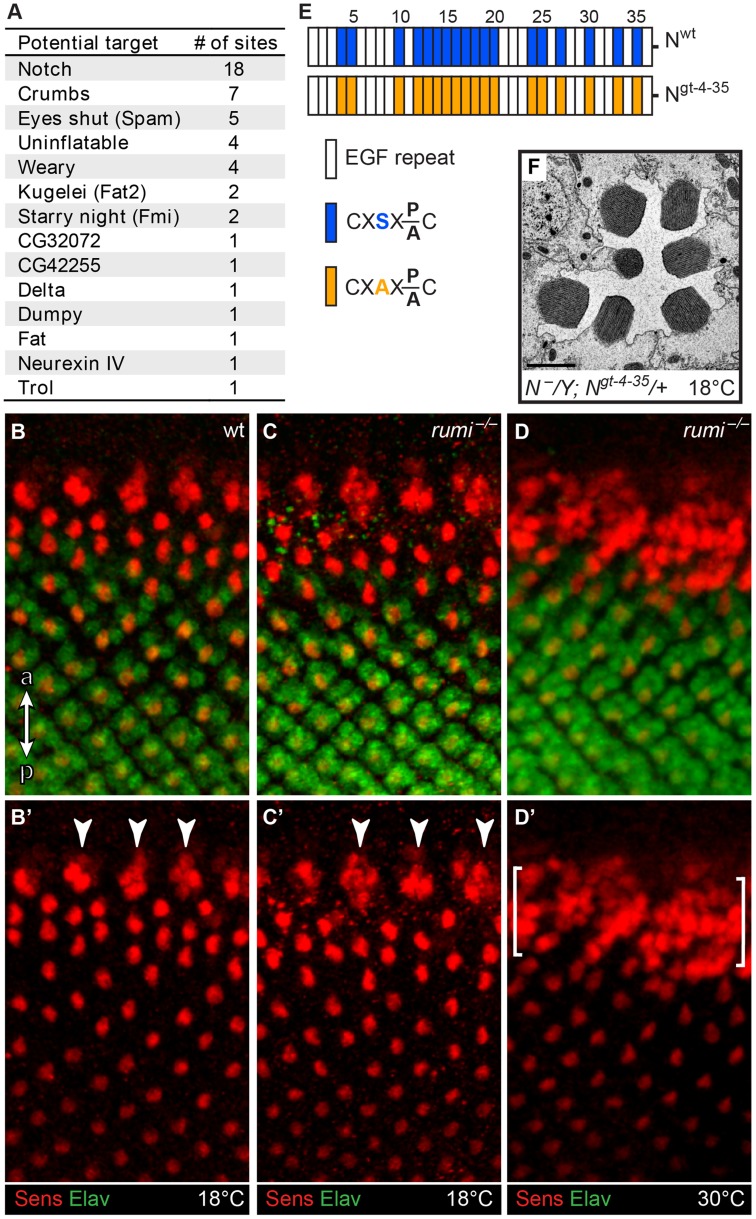
Loss of *O*-glucose on Notch cannot explain the *rumi^−/−^* rhabdomere attachment phenotype. (A) List of potential Rumi targets in flies. (B–D′) Close-ups of the third instar larval eye discs showing the developing photoreceptors in wt (B,B′), *rumi^−/−^* animals raised at 18°C (C,C′) and *rumi^−/−^* animals raised at 18°C and shifted to 30°C for four hours before dissection (D,D′). R8 photoreceptors are marked with Senseless (Sens, red) and all photoreceptors are marked with Elav (green). ‘a’ and ‘p’ in panel B show anterior and posterior, respectively, and apply to B–D′. Arrowheads in B′ and C′ mark the R8 proneural clusters before a single R8 is selected through Notch-mediated lateral inhibition. In *rumi^−/−^* animals raised at 18°C (C,C′), the R8 proneural clusters are refined into single Sens^+^ R8 cells, which themselves recruit other photoreceptors, similar to the wild-type animal (B,B′). In contrast, raising the *rumi* larvae at 30°C results in the specification of multiple R8 photoreceptor cells (the area between the brackets), indicating an impairment of the Notch-mediated lateral inhibition at this temperature. Note that R8 selection and photoreceptor recruitment is not impaired in the area posterior to the brackets because the animal was raised at 30°C only for four hours. (E) Schematic of the EGF repeats of the Notch genomic transgene used in the study. Blue and orange boxes indicate EGF repeats harboring wt and mutant Rumi consensus sequences, respectively. (F) Electron micrograph of an ommatidium of an animal expressing the mutated *PBac{Notch^gt-4-35^}* transgene in a *Notch* null background raised at 18°C. Scale bar: 2 µm.

If Rumi regulates rhabdomere spacing via its protein *O*-glucosyltransferase (Poglut) activity, lack of glucose on Rumi target proteins is likely to be responsible for the observed phenotype. To identify all fly proteins with a potential Rumi target site, we used the MOTIF search engine (http://motif.genome.jp/MOTIF2.html) to search the Swiss-Prot and KEGG-GENES databases for *Drosophila* proteins harboring one or more EGF repeats with the C^1^XSX(P/A)C^2^ consensus sequence [13]. Based on this search, 14 *Drosophila* proteins have at least one EGF repeat with a predicted Rumi target site (Figure 2A), with Notch harboring the largest number of *O*-glucosylation sites, most of which have been confirmed to be efficiently *O*-glucosylated by Rumi [12], [13], [18]. *rumi* null animals raised at 18°C do not show photoreceptor specification defects characteristic of loss of Notch signaling (Figure 2B–D′), suggesting that Notch signaling is not significantly affected in *rumi^−/−^* developing photoreceptors at this temperature. Moreover, to our knowledge, Notch signaling has not been implicated in rhabdomere spacing. Nevertheless, given the broad roles that Notch plays in multiple developmental contexts, we sought to examine whether the rhabdomere spacing defects observed in *rumi* mutants can be explained by loss of *O*-glucose from Notch EGF repeats. To this end, we used a *Notch* genomic transgene (*PBac{N^gt-4-35^}*) in which serine-to-alanine mutations are introduced in all 18 Rumi target sites and therefore expresses a Notch protein which cannot be *O*-glucosylated by Rumi [14] (Figure 2E). When reared at 18°C, the *PBac{N^gt-4-35^}* transgene rescues the lethality of *Notch* null mutations, and the *N^−^; PBac{N^gt-4-35^}/+* animals only show a mild loss of Notch signaling similar to *rumi* mutants [14]. TEM revealed that adult *N^−^; PBac{N^gt-4-35^}/+* eyes raised at 18°C do not show any rhabdomere attachment phenotypes (Figure 2F), strongly supporting the notion that addition of *O*-glucose to Notch is not essential for proper rhabdomere spacing.

### Crb is *O*-glucosylated but loss of *O*-glucose from Crb does not result in rhabdomere attachment

**Figure 3:**
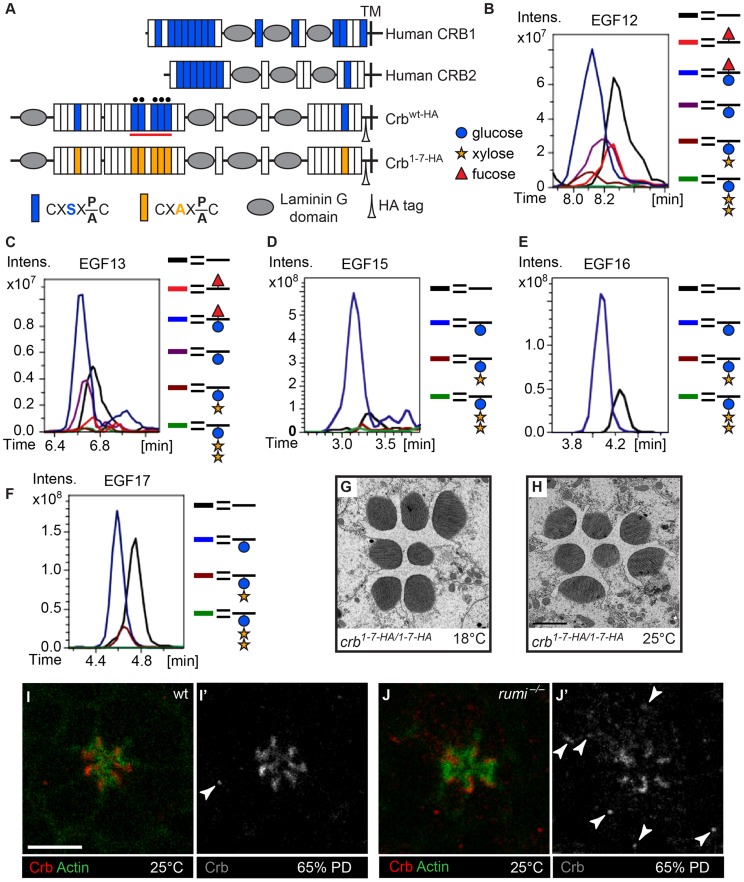
Loss of *O*-glucose on Crb cannot explain the *rumi^−/−^* rhabdomere attachment phenotype. (A) Schematic of the human CRB1, human CRB2, and wt and mutant, HA-tagged *Drosophila* Crb based on the Crb-PA polypeptide (FlyBase ID: FBpp0083987). Blue and orange boxes indicate EGF repeats harboring wt and mutant Rumi consensus sequences, respectively. TM, transmembrane domain. Black circles mark EGF repeats shown in B–F. (B–F) Extracted Ion Chromatogram (EIC) data for peptides containing the *O*-glucose consensus site from Crb EGF12 (B), EGF13 (C), EGF15 (D), EGF16 (E) and EGF17 (F) obtained from mass spectrometry on a Crb fragment indicated by the red line in (A). Note the presence of *O*-glucose (blue circle) on all five EGF repeats, some of which are elongated by xylose (yellow star). Peptides from EGF12 and EGF13 also harbor *O*-fucose (red triangle) added to the consensus *O*-fucosylation sites present in these two EGF repeats. Full spectra and the peptide sequences are shown in Figure S2 and Figure S3. (G,H) *crb* knock-in mutants lacking all Rumi target sites and raised at 18°C (G) or 25°C (H) do not show rhabdomere attachment. Scale bar in H applies to G and H and is 2 µm. (I–J′) Loss of Rumi does not impair Crb localization to the stalk membrane. Increased Crb puncta, some of which are marked with arrowheads in J′, are seen in the photoreceptor cell body of *rumi^−/−^* ommatidia raised at 25°C (J,J′) compared to wild-type (I,I′). Actin (green) is used to mark the rhabdomeres. Crb is shown in red. Scale bar: 5 µm.

The fly protein with the second largest number of Rumi target sites is Crb (Figure 2A), an evolutionarily conserved transmembrane protein involved in the regulation of epithelial polarity, organ size, and photoreceptor development and maintenance [10], [11], [20]–[24]. Of note, *crb* mutant retinas exhibit attachment of neighboring rhabdomeres despite the presence of IRS [20], [21]. Seven of the *Drosophila* Crb EGF repeats, 13 of the human CRB1 EGF repeats and eight of the human CRB2 EGF repeats harbor Rumi target sites, suggesting that *O*-glucosylation might play an important role in the function of Crb (Figure 3A). We performed mass spectral analysis on peptides derived from a fragment of the Crb extracellular domain expressed in *Drosophila* S2 cells (Figure 3A, the red line) to examine whether Crb can be *O*-glucosylated in *Drosophila*. Indeed, peptides containing the predicted sites in this region are *O*-glucosylated (Figure 3B–F, Figure S2 and Figure S3). We next asked whether loss of *O*-glucose from Rumi target sites in Crb recapitulates the rhabdomere attachment phenotype observed in *rumi* mutants. Using a previously established platform [23], [25], [26], we generated a knock-in allele of *crb* (*crb^1-7-HA^*) with serine-to-alanine mutations in all seven Rumi target sites (Figure 3A). Animals homozygous for this allele or trans-heterozygous for this allele and the null allele *crb^11A22^* are viable and do not exhibit any gross abnormalities when raised between 18°C and 25°C. Moreover, TEM indicates normal rhabdomere morphology and IRS formation with no defects in rhabdomere spacing in *crb^1-7-HA/1-7-HA^* animals raised at either 18°C or 25°C (Figure 3G and 3H). These observations indicate that absence of Crb *O*-glucosylation does not explain the rhabdomere spacing defects of *rumi* mutants. In agreement with these data, Crb appears to be properly localized to the stalk membrane in 65% PD *rumi^−/−^* retinas, although an increase in the number of Crb^+^ puncta is seen in *rumi* mutants raised at 25°C compared to control animals (Figure 3I–J′, arrowheads). Together, these data indicate that although *O*-glucose modifications might affect the trafficking of Crb, they are not essential for the function of Crb during fly embryonic development and photoreceptor morphogenesis.

### Eys is a biologically-relevant target of Rumi during rhabdomere morphogenesis

**Figure 4:**
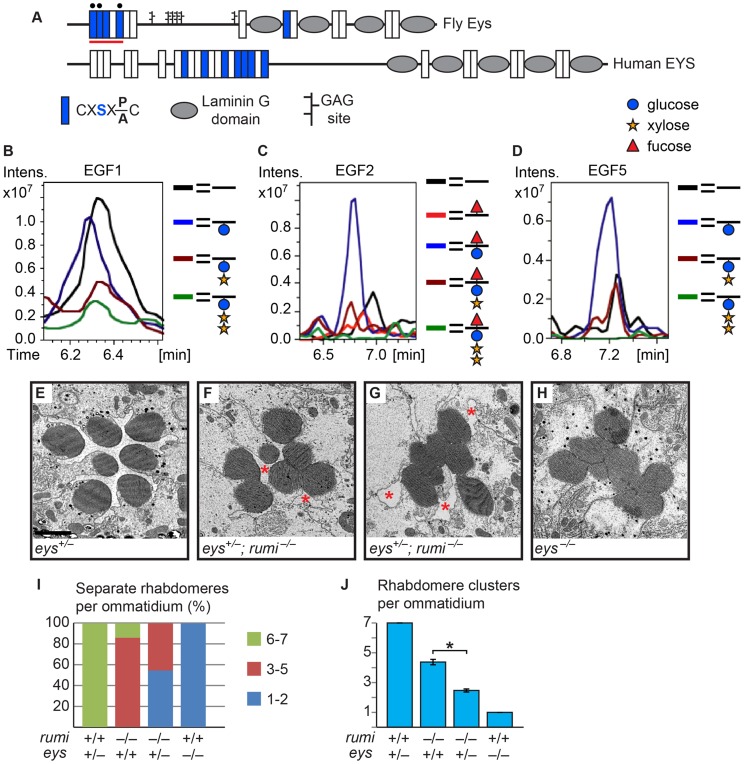
Eys is *O*-glucosylated and *eys* genetically interacts with *rumi*. (A) Schematic of the fly and human Eys proteins. Red line indicates the EGF repeats used for mass spectrometry analysis. Black circles mark EGF repeats shown in B–D. (B–D) EIC data from mass spectral analysis of EGF1 (B), EGF2 (C), and EGF5 (D). Blue peak indicates the addition of *O*-glucose (blue circle) onto the EGF repeat. EGF2 is also modified by *O*-fucose (red triangle). Rumi appears to be less efficient in *O*-glucosylating EGF1 of Eys compared to the other EGF repeats when expressed in S2 cells. Full spectra and the peptide sequences are shown in Figure S4. (E) An *eys* heterozygous ommatidium with normal rhabdomere separation. Scale bar: 2 µm. (F,G) *eys^+/−^ rumi^−/−^* ommatidia show a dramatic enhancement of the *rumi^−/−^* phenotype. In severe cases, all the rhabdomeres are attached (G). Pockets of IRS are marked by asterisks. (H) *eys* null ommatidia completely lack IRS. (I) Percentage of number of individual rhabdomeres per ommatidium for various genotypes. At least three animals were used for each genotype. (J) Quantification of average individual rhabdomere number per ommatidium for the data shown in I. All pairs are significantly different from each other (*P<0.0001).

As mentioned above, another *Drosophila* protein with multiple predicted Rumi target sites and an IRS phenotype is Eys (Figure 2A and Figure 4A) [5], [6]. To examine whether Eys is the biologically-relevant target of Rumi in the context of rhabdomere spacing, we first performed mass spectral analysis on peptides derived from an Eys fragment harboring four Rumi target sites expressed in S2 cells (Figure 4A, the red line). So far we have been able to identify peptides corresponding to three of these sites by mass spectrometric analysis and have identified *O*-glucose on all three sites (Figure 4B–D and Figure S4). The Rumi target site in EGF1 appears to be less efficiently *O*-glucosylated compared to those in other Eys EGF repeats. Nevertheless, these data indicate that *Drosophila* Eys contains several bona fide Rumi targets. We next performed genetic interaction studies between *rumi* and *eys* by using the protein-null allele *eys^734^* [5]. As reported previously, loss of one copy of *eys* does not cause any rhabdomere defects (Figure 4E) [5], [6]. However, removing one copy of *eys* in a *rumi^−/−^* background results in a strong enhancement of the *rumi^−/−^* rhabdomere attachment phenotype at 18°C (Compare Figure 4F and 4G to Figure 1B, 1D and 1E). In *eys^+/−^; rumi^−/−^* animals, multiple rhabdomeres collapse into one another in each ommatidium, and there is a dramatic decrease in the IRS size (Figure 4F, 4G, 4I and 4J). Of note, pockets of IRS can still be recognized in all *eys^+/−^; rumi^−/−^* ommatidia (Figure 4F and 4G, asterisks), in contrast to *eys^−/−^* ommatidia, which completely lack IRS (Figure 4H and 4J) [5]. This dosage-sensitive genetic interaction strongly suggests that Rumi is critical for the function of Eys, especially when Eys levels are limiting.

### Loss of Rumi results in a decrease in the extracellular level of Eys in a temperature-dependent manner

**Figure 5:**
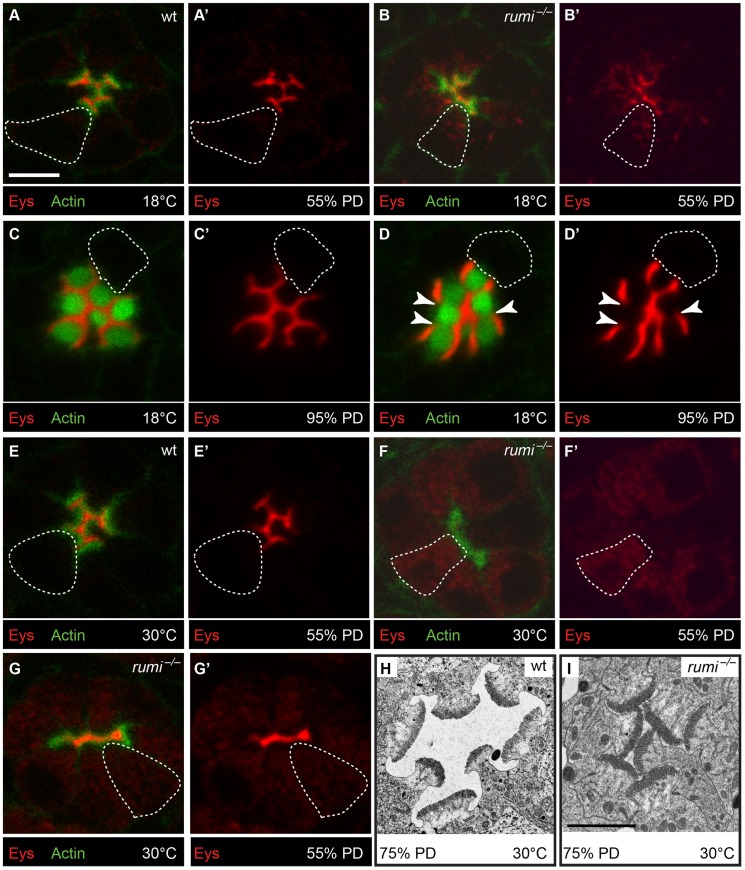
Loss of Rumi leads to intracellular accumulation and decreased IRS levels of Eys in a temperature dependent manner at the mid-pupal stage. (A–G′) Confocal micrographs each showing a single ommatidium from the indicated genotypes. Phalloidin (green) marks Actin and is concentrated in rhabdomeres; Eys is shown in red. The dotted shapes mark the outline of a single photoreceptor cell body in each micrograph. The scale bar in A applies to A–G′ and is 5 µm. (A,A′) A wild-type ommatidium at 55% PD raised at 18°C. Note that Eys is primarily localized to the IRS. (B,B′) A *rumi^−/−^* ommatidium at 55% PD raised at 18°C. Note the decreased level of Eys in the IRS and its accumulation in the cell body. (C,C′) A wild-type ommatidium at 95% PD raised at 18°C. (D,D′) A *rumi^−/−^* ommatidium at 95% PD raised at 18°C. Note the increased amount of Eys in the IRS at this stage and disappearance of Eys from the photoreceptor cell bodies compared to B. White arrowheads mark points of rhabdomere attachment and gaps in Eys. (E,E′) A single ommatidium from a wild-type animal at 55% PD shifted to 30°C during IRS formation. (F–G′) Ommatidia from *rumi^−/−^* animals at 55% PD shifted to 30°C during IRS formation show severe Eys accumulation in the cell body, with either a complete lack of Eys in the IRS (F,F′) or a thin line of Eys in the IRS (G,G′). (H,I) Electron micrographs showing wild-type (H) and *rumi^−/−^* (I) ommatidia from 75% PD animals shifted to 30°C during IRS formation. The scale bar in I applies to H and I and is 2 µm.

Secretion of Eys from the apical surface of the photoreceptor cells at the mid-pupal stage separates the rhabdomeres from one another and generates the IRS [5], [6]. Based on the modENCODE Temporal Expression Data accessed on FlyBase (http://flybase.org/reports/FBgn0031414.html), expression of *eys* sharply increases at mid-pupal stage and gradually decreases in later pupal stages. Following the initial burst of Eys expression between 50–70% PD [5] and rhabdomere separation, Eys continues to be secreted into the IRS, which gradually enlarges and assumes its adult size late in the pupal stage [5], [19]. Given the increased degree of rhabdomere attachment and the severe decrease in the IRS size in adult animals simultaneously lacking *rumi* and one copy of *eys*, we examined whether loss of Rumi affects Eys levels in the IRS. We first compared Eys expression in the early stages of IRS development between *rumi^−/−^* and control pupae raised at 18°C. At 55% PD, the rhabdomeres of control animals are separated from one another by a thin but continuous IRS filled with Eys, and only low levels of Eys can be detected in photoreceptor cell bodies (Figure 5A and 5A′) [5]. In contrast, *rumi^−/−^* ommatidia almost invariably show some degree of rhabdomere attachment and a decreased and interrupted pattern of Eys expression in the IRS (Figure 5B and 5B′). In the majority of *rumi^−/−^* ommatidia examined, decreased levels of Eys in the IRS are accompanied by increased Eys levels in the photoreceptor cell bodies (Figure 5B and 5B′). Quantification of the total pixel intensity of Eys at 55% PD in animals raised at 18°C shows that in wild-type ommatidia, 87.1±2.0% of total Eys is found in the IRS and the rest is in photoreceptor cell bodies. However, there is a statistically significant decrease in the percentage of Eys found in the IRS in *rumi^−/−^* ommatidia (63.6±6.5%, *P* = 0.01). These data indicate that during early stages of IRS formation, a significant amount of Eys remains inside the photoreceptor cells in *rumi* mutants, unlike wild-type animals, in which most of the Eys is efficiently secreted into the IRS.

As shown above, *rumi* mutants raised at 18°C show rhabdomere attachment and a significantly decreased IRS size in the mid-pupal stage (Figure 1K). However, in *rumi^−/−^* adults, even though the rhabdomere attachments persist, the IRS in the center of the ommatidia looks similar to that in control ommatidia (Figure 1A–E), suggesting that enough Eys is secreted in later pupal stages to expand the IRS. To test this notion, we examined Eys expression in wild-type and *rumi* null animals at 95% PD. In wild-type animals, Eys fills the IRS in an uninterrupted manner and cannot be seen in the cell body (Figure 5C–C′). In *rumi* mutants, Eys is properly localized to the IRS at levels similar to that found in wild-type IRS and is not visible in the cell body (Figure 5D and 5D′). However, multiple gaps in the Eys expression domain are seen in the IRS, coinciding with rhabdomere attachments (Figure 5D and 5D′, white arrowheads). These data suggest that the rhabdomere attachments in *rumi* mutants result from decreased levels of Eys in the IRS in a critical period during the mid-pupal stage and that these attachments are not resolved later in pupal development despite continued secretion of Eys.

Since the loss of Notch signaling in *rumi* mutants is temperature-sensitive [12], [14], [15], we next examined whether the IRS defect observed in *rumi* animals becomes worse at higher temperatures. To bypass the larval lethality and photoreceptor specification defects of *rumi* mutants at 30°C, we kept *rumi* mutant and control animals at 18°C until the end of the third instar stage and shifted them to 30°C at zero hours after puparium formation (APF) so that they were kept at high temperature at mid-pupal stage, when *eys* expression starts [5]. However, these animals died by mid-pupal stage, precluding the study of Eys secretion and IRS formation. Therefore, we modified our temperature shift regimen by transferring *rumi^−/−^* and control animals to 25°C at zero h APF, shifted them to 30°C at 24 h APF and kept them at this temperature until 55%–75% pupal development, when we dissected them for staining or TEM. The patterns of Phalloidin and Eys staining in control animals looked similar to those raised at 18°C (Figure 5E and 5E′). In contrast, *rumi* mutants either lacked Eys in the IRS (Figure 5F and 5F′) or had small Eys-containing regions (Figure 5G and 5G′). Most *rumi* mutant ommatidia showed high levels of Eys in the photoreceptor cells (Figure 5F–G′). TEM on *rumi^−/−^* animals reared under the above mentioned conditions showed multiple sites of rhabdomere attachment and a small IRS at 75% PD compared to control animals raised under the same conditions (Figure 5H and 5I). These observations indicate that in *rumi* mutants grown at higher temperatures, a higher fraction of Eys remains inside the cell and the level of Eys in the IRS is further reduced.

**Figure 6:**
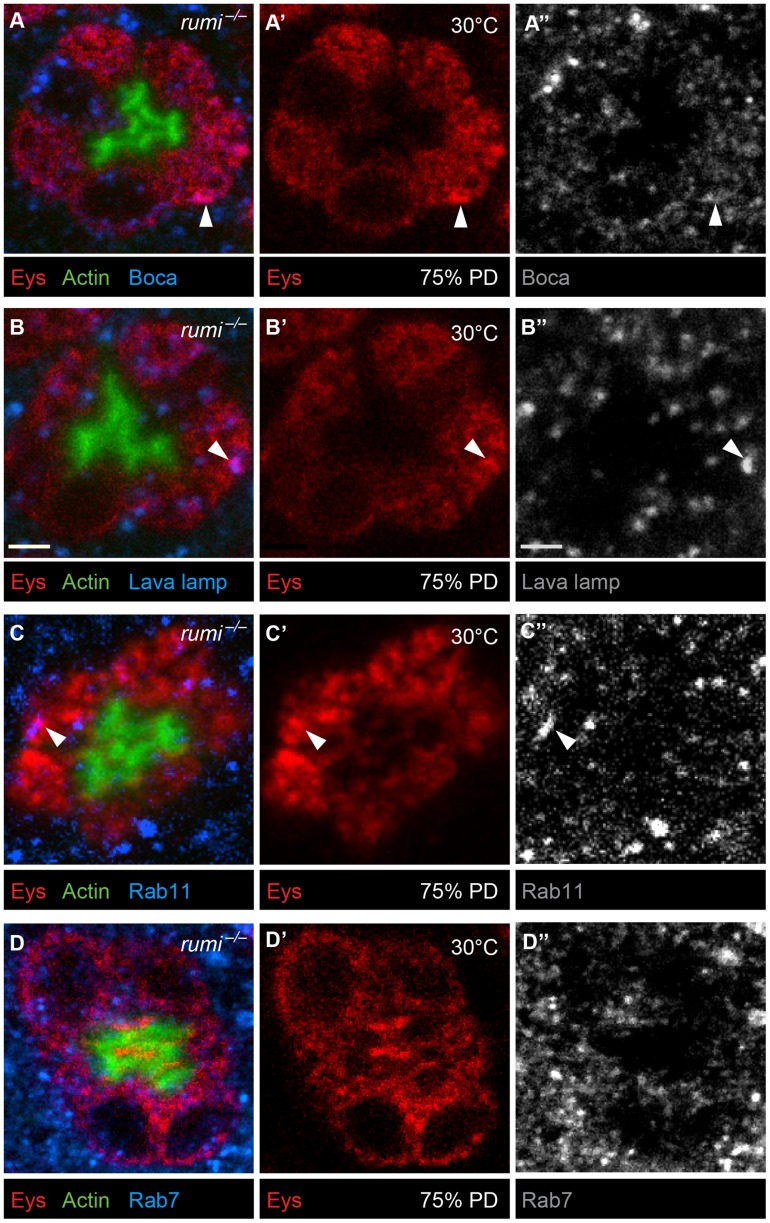
Eys does not accumulate in a single subcellular compartment in *rumi^−/−^* photoreceptors raised at 30°C. Confocal micrographs from 75% PD *rumi^−/−^* ommatidia colabeled with Eys and the ER marker Boca (A–A″), the Golgi marker Lava lamp (B–B″), the recycling endosomal marker Rab11 (C–C″), and the late endosomal marker Rab7 (D–D″). White arrowheads mark colocalization between Eys and the respective subcellular compartment. Eys only shows occasional colocalization with each marker, strongly suggesting that Eys does not accumulate in a single compartment.

To examine whether Eys accumulates in a specific subcellular compartment in *rumi* mutant photoreceptor cells, we performed colocalization studies between Eys and markers of ER (Figure 6A–A″), Golgi (Figure 6B–B″), recycling endosome (Figure 6C–C″), and the late endosome (Figure 6D–D″) in *rumi* null animals shifted to 25°C at zero h APF and later to 30°C at 24 h APF as explained above. Eys was transported to all cellular compartments examined, as shown by occasional colocalization with each marker (Figure 6A–D″, white arrowheads), indicating that Eys trafficking is not blocked at a single step in the secretion pathway but is likely slowed down through the secretory pathway, causing it to accumulate in the cell body as it travels to the membrane.

**Figure 7:**
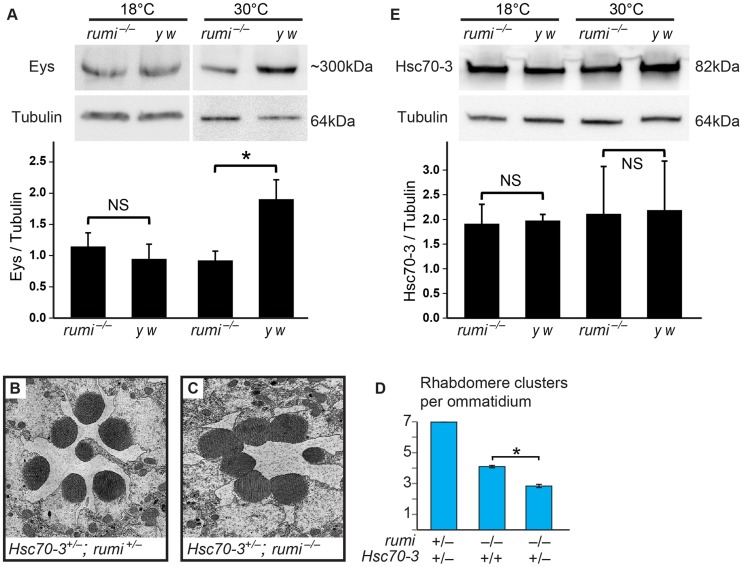
*rumi^−/−^* animals show a temperature-dependent decrease in Eys levels and a dosage-sensitive genetic interaction with an ER chaperone. (A) Western blots showing Eys levels in *rumi^−/−^* and *y w* heads grown at 18°C (left) and 30°C (right). There is a statistically significant decrease in Eys levels in *rumi^−/−^* heads raised at 30°C (*P*<0.05; lower panel). (B,C) *Hsc70-3^+/−^; rumi^−/−^* ommatidia show an enhancement of the *rumi^−/−^* phenotype. (B) Electron micrograph showing a single ommatidium from an *Hsc70-3^+/−^; rumi^+/−^* control animal. (C) Electron micrograph showing an *Hsc70-3^+/−^; rumi^−/−^* ommatidium. Frequently, all rhabdomeres but one are attached. (D) Quantification of average individual rhabdomere number per ommatidium for the indicated genotypes. *P<0.0001. (E) Levels of Hsc70-3, which is induced upon UPR, do not change between *rumi^−/−^* and *y w* animals raised at different temperatures. Top panel: western blot showing Hsc70-3 levels and Tubulin loading control. Bottom panel: ratio of Hsc70-3/Tubulin levels, which do not change between genotypes. NS, not significant.

Worsening of the IRS defect and further decrease in the extracellular levels of Eys in *rumi* mutant animals raised at higher temperature suggest that in the absence of Rumi, Eys is misfolded. To assess the effects of loss of Rumi on Eys protein levels at low and high temperatures, we performed Western blots on head extracts from 80% PD wild-type and *rumi^−/−^* animals. When raised at 18°C throughout development, wild-type and *rumi^−/−^* pupae did not show a significant difference in the level of Eys (Figure 7A, left panel, *P* = 0.57). However, when the animals were raised at 18°C until mid-pupal stage and shifted to 30°C during IRS formation, there was a significant decrease in the level of Eys in *rumi^−/−^* pupal heads (Figure 7A, right panel, *P*<0.05). These data support the notion that loss of Rumi decreases the ability of Eys to fold properly and to be secreted at a normal rate. The data also suggest that at higher temperatures, misfolding results in degradation of Eys and worsening of IRS defects in *rumi* mutant animals.

If rhabdomere attachments observed in *rumi* mutants result from Eys misfolding, decreasing the level of chaperone proteins might enhance this phenotype. To test this hypothesis, we examined whether removing one copy of the ER chaperone *Hsc70-3* (BiP) affects rhabdomere attachment in *rumi^−/−^* animals. As shown in Figure 7B, animals double heterozygous for a lethal *P*-element inserted in the coding region of *Hsc70-3* (*Hsc70-3^G0102^*) and *rumi* do not exhibit any rhabdomere attachment. However, *Hsc70-3^G0102/+^; rumi^−/−^* animals raised at 18°C show an enhancement of the rhabdomere attachment phenotype observed in *rumi^−/−^* animals raised at the same temperature (Figure 7C; compare to Figure 1). The average number of separate rhabdomeres in *Hsc70-3^G0102/+^; rumi^−/−^* animals is significantly different from *rumi^−/−^* animals (Figure 7D, 2.85±0.11 vs. 4.11±0.08, *P*<0.0001). This observation further supports the conclusion that Eys is misfolded in *rumi* mutants. We next asked whether loss of Rumi triggers the unfolded protein response (UPR) in the pupal eye. One of the hallmarks of the UPR is the induction of chaperones, including Hsc70-3 [27]. Western blotting using anti-Hsc70-3 antibody did not show an increase in the level of Hsc70-3 expression in *rumi* mutants raised at 18°C or 30°C compared to control animals (Figure 7E). This indicates that UPR is not induced in the pupal eyes upon loss of Rumi, in agreement with a previous report on lack of UPR induction in *rumi^−/−^* clones in wing imaginal discs raised at 28°C despite accumulation of the Notch protein [12].

### Mutations in Eys *O*-glucosylation sites result in its intracellular accumulation

**Figure 8:**
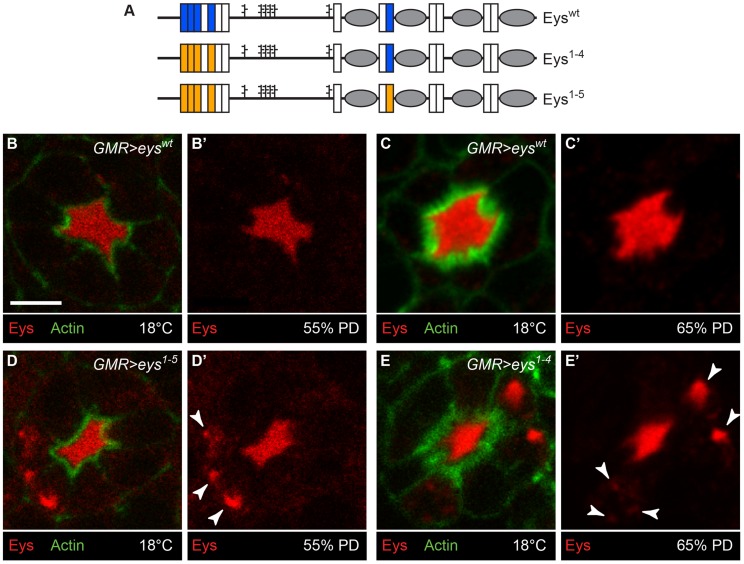
Loss of *O*-glucose on Eys results in its intracellular accumulation. (A) Schematic of Eys^wt^, Eys^1-4^, in which four out of five Rumi target sites are mutated, and Eys^1-5^, in which all Rumi target sites are mutated. (B–E′) Overexpression of Eys^1-5^ (D,D′) and Eys^1-4^ (E,E′), but not Eys^wt^ (B–C′), results in intracellular Eys accumulation (arrowheads). The *GMR-GAL4* driver was used and all animals were raised at 18°C. Note that the *GMR>eys^wt^* animal shown in (B,B′) and the *GMR>eys^1-5^* animal (D,D′) are younger than the *GMR>eys^wt^* animal shown in (C,C′) and the *GMR>eys^1-4^* animal (E,E′). Scale bar: 5 µm.

If loss of the Poglut activity results in intracellular accumulation of Eys in the mid-pupal stage, mutating the Rumi target sites on Eys should affect its trafficking as well. To test this, we generated *UAS-attB* transgenes capable of overexpressing wild-type Eys (Eys^wt^) or Eys with serine-to-alanine mutations in four (Eys^1-4^) or in all five Rumi target sites (Eys^1-5^) (Figure 8A). To minimize the expression variability associated with random insertion of transgenes, we used ΦC31 transgenesis and integrated all three constructs in the same docking site (*VK31*) in the fly genome [28], [29]. We used *GMR-GAL4* to overexpress wild-type and mutant Eys in the developing photoreceptors and kept the animals at 18°C to avoid the very high levels of GAL4-driven transgene expression at high temperatures. In animals overexpressing wild-type Eys, the IRS is expanded and the majority of the Eys is within the IRS, although low levels of Eys are seen in photoreceptor cells (Figure 8B–C′). Overexpression of Eys^1-4^ and Eys^1-5^ also expands the IRS (Figure 8D–E′, compare to Figure 5). However, unlike the wild-type protein, *O*-glucose mutant versions of Eys protein accumulate in the photoreceptor cells (Figure 8D–E′, white arrowheads). These data support a role for *O*-glucose residues in the proper folding and trafficking of Eys.

## Discussion

We have previously shown that the extracellular domains of *Drosophila* and mammalian Notch proteins are efficiently *O*-glucosylated, and have provided strong evidence that Rumi/POGLUT1 is the only protein *O*-glucosyltransferase capable of adding *O*-glucose to EGF repeats in animals [12], [13], [16], [18], [30]. The data presented here indicate that *Drosophila* Crb and Eys also harbor *O*-glucose residues, yet the impact of loss of Rumi and loss of *O*-glucose from these three target proteins, which harbor the highest number of Rumi target sites among all *Drosophila* proteins, is not equivalent. Loss of Rumi and mutations in Rumi target sites in a *Notch* genomic transgene both result in a temperature-dependent loss of Notch signaling [12], [14], indicating that the Notch protein becomes sensitive to temperature alterations in the absence of *O*-glucose. Although the Notch loss-of-function phenotypes in *rumi* mutants raised at 18°C are mild and limited to certain contexts, raising animals homozygous for *rumi* or harboring *rumi* mitotic clones at 28–30°C phenocopies *Notch*-null phenotypes [12], [15], [17], indicating that *O*-glucose is indispensable for the function of *Drosophila* Notch at the restrictive temperature. At a functional level, loss of Rumi affects Eys similarly, with a moderate rhabdomere attachment phenotype at 18°C which becomes more severe when *rumi* animals are raised at 30°C during the IRS formation. However, even when raised at 30°C, *rumi* does not phenocopy an *eys*-null phenotype in the eye, as rhabdomeres show some degree of separation in the mid-pupal stage. The function of Crb, in contrast, does not seem to be significantly affected by loss of *O*-glucose, as flies homozygous for a mutant allele of *crb* which contains no intact Rumi consensus sequences are viable and fertile, and do not exhibit any obvious phenotypes in rhabdomere morphogenesis. The divergent effects of *O*-glucose on the function of these proteins does not seem to be correlated with the number of Rumi target sites or the overall structure of these proteins, as Notch and Crb are transmembrane proteins but Eys is secreted, Crb and Eys both have a combination of EGF repeats and Laminin G domains but Notch does not have Laminin G domains, and Crb has a higher number of Rumi target sites (seven) compared to Eys (five). In summary, our data indicate that although the C^1^XSX(P/A)C^2^ motif is highly predictive for the addition of *O*-glucose to EGF repeats of *Drosophila* proteins, the functional importance of *O*-glucose depends on additional parameters beyond the number of *O*-glucose sites and the overall domain structure of a given target protein.

In *rumi* mutant ommatidia, a significant amount of Eys remains inside the photoreceptor cells, while the extracellular levels of Eys in the IRS decrease. At the restrictive temperature, these phenotypes are enhanced and the total level of Eys in *rumi* mutant heads is significantly decreased. Moreover, removing one copy of an important ER chaperone enhances the rhabdomere attachment phenotype in *rumi* mutants. Finally, animals homozygous for the catalytically-inactive allele *rumi^79^* also show rhabdomere attachment, and mutating the *O*-glucose sites of Eys results in its intracellular accumulation. Together, these observations strongly suggest that loss of *O*-glucosylation results in Eys misfolding and a decrease in its extracellular levels. In contrast, despite the almost complete loss of Notch signaling in *rumi* clones raised at 28–30°C, surface expression of Notch is not decreased upon loss of Rumi; indeed, Notch accumulates inside and at the surface of *rumi* mutant epithelial cells raised at the restrictive temperature [12]. Moreover, cell-based and genetic experiments suggest that in the absence of Rumi, Notch is able to bind ligands at the cell surface but fails to be cleaved properly by the ADAM10 metalloproteinase Kuzbanian [12], [14]. Therefore, although these reports cannot rule out a redundant role for *O*-glucose in promoting the cell surface expression of Notch, they indicate that *O*-glucose is required for Notch signaling independently of its exocytic trafficking. Nevertheless, the temperature-dependent enhancement of loss of Notch signaling and Notch accumulation in *rumi* mutants [12], [14] suggests that folding of Notch might also be affected by the loss of *O*-glucose. Similarly, the increase in the number of Crb^+^ puncta observed in *rumi^−/−^* photoreceptors raised at 25°C suggests that although the function of *Drosophila* Crb does not depend on *O*-glucosylation, loss of *O*-glucose affects Crb trafficking. Therefore, while we cannot rule out that *O*-glucosylation affects each of these targets differently at molecular and cell biological levels, we favor a scenario in which the folding of all three targets is affected by loss of *O*-glucose. In this scenario, the degree of functional defects observed for each target and the cellular compartment where the defect is observed varies depending on the extent of misfolding, the sensitivity of the target protein to lack of *O*-glucose and the cellular context where the target operates. It is intriguing to note that Rumi/POGLUT1 only glucosylates properly folded EGF repeats *in vitro* [31], suggesting that Rumi/POGLUT1 may exert its effects on folding at the level of individual EGF repeats.

Analysis of rhabdomere separation and IRS size in mid-pupal and late pupal/adult *rumi^−/−^* animals raised at 18°C suggests two temporally distinct steps for the function of Eys during IRS formation. In the early stages of IRS formation, some of the rhabdomeres in each ommatidium fail to separate from each other, and the mutant IRS is significantly smaller than control IRS. In late pupal stages, the level of Eys in the IRS of *rumi^−/−^* ommatidia is comparable to that in control ommatidia, in agreement with the more or less normal IRS size observed in adult *rumi* ommatidia. Nevertheless, rhabdomere attachments are not resolved. These observations suggest that Eys generates the IRS in two steps. At ∼45–55% PD, Eys secretion is required to sever the attachments among the rhabdomeres in each ommatidium (step 1), likely by opposing the adhesive forces mediated by Chaoptin [6]. Rhabdomere separation in turn generates conduits between stalk membranes—where Eys is secreted [5] —and the central IRS, and thereby allows Eys to increase the IRS size after rhabdomeres are separated (step 2). We propose that in *rumi* mutants, the Eys protein fails to fold properly and as a result, a significant fraction of Eys remains inside the cell instead of being secreted into the extracellular space. Therefore, at the mid-pupal stage Eys fails to fully separate the rhabdomeres from one another. Once the critical time window between 45–55% PD (step 1) passes, continued Eys secretion in step 2 (IRS expansion) cannot separate rhabdomeres anymore. However, since in each *rumi* ommatidium some rhabdomeres separate from one another, Eys can reach the central IRS and can gradually increase the IRS size. This two-step model of rhabdomere separation and IRS expansion is further supported by the observation that overexpression of Eys in an *eys* null background after 65% PD fails to separate the rhabdomeres [6].

Lack of photoreceptor abnormalities in *crb* mutants with no intact Rumi target sites was somewhat surprising, given that Crb has the second highest number of *O*-glucosylation motifs in all fly proteins and that multiple EGF repeats in human CRB1 and CRB2 contain the Rumi consensus sequence. Our data indicate that *O*-glucosylation of Crb is not required for viability, fertility and photoreceptor morphogenesis in flies, at least in a laboratory setting. The Crb extracellular domain is dispensable for proper apical-basal polarity in embryos [32], but is required in other contexts, such as stalk membrane formation [11], regulation of the head size [24], prevention of light-induced photoreceptor degeneration [21] and invagination of the salivary gland placode in embryos [33]. While the stalk membrane formation is not impaired upon mutating all Crb *O*-glucose sites, it remains to be determined whether *O*-glucosylation of Crb is required for the regulation of other processes regulated by the Crb extracellular domain, and whether *O*-glucosylation of mammalian CRB proteins is required for their function.

Although a number of mammalian species including mice, rats, guinea pigs and sheep have lost Eys during evolution [34], humans have an Eys homolog (EYS), which shows an overall protein domain organization similar to the fly Eys (Figure 4) [35]. Transgenic expression of human EYS in a *Drosophila eys* null background produces pockets of IRS, presumably at the location of secretion, but fails to rescue the rhabdomere attachment phenotype [9]. However, when human EYS is coexpressed in *eys^−/−^* animals with a human homolog of the *Drosophila* Prom called PROM1, some rhabdomeres separate from their neighbors [9]. Since binding between *Drosophila* Eys and Prom is important for IRS formation [6], these rescue experiments highlight the evolutionary conservation of the Eys-Prom interaction in the visual system. Of note, mutations in human *EYS* and *PROM1* cause several forms of retinal degeneration including autosomal recessive retinitis pigmentosa, rod-cone dystrophies and cone-rod dystrophy [34]–[43]. Human EYS contains seven target sites for *O*-glucosylation, 4–5 of which are clustered similar to the Rumi target sites in EGF1-5 of *Drosophila* Eys. Therefore, *O*-glucosylation might play an important role in the function of the human EYS.

## Materials and Methods

### *Drosophila* strains and genetics

The following strains were used in this study: 1) *Canton-S*, 2) *y w*, 3) *w; noc^Sco^/CyO; TM3, Sb^1^/TM6, Tb^1^*, 4) *y w; D/TM6, Tb^1^*, 5) *N^55e11^/FM7c*, 6) y^1^ w^67c23^ P{Crey}1b; D^*^/TM3, Sb^1^, 7) y^1^ M{vas-int.Dm}ZH-2A w^*^; VK31, 8) y^1^ M{vas-int.Dm}ZH-2A w^*^; VK22, 9) *w^67^ c^23^ P{lacW}Hsc70-3^G0102^/FM7c*, 10) *GMR-GAL4* (on 2) (Bloomington *Drosophila* Stock Center), 11) *GMR-GAL4* (on 3) [44], 12) *eys^734^* [5], 13) *P{rumi^gt-FLAG^}* (*rumi* rescue transgene), 14) *y w; FRT82B rumi^79^/TM6, Tb^1^*, 15) *y w; FRT82B rumi^Δ26^/TM6, Tb^1^* [12], 16) *y w; PBac{N^gt-4-35^}attVK22* [14], 17) *eys^734^/CyO; FRT82B rumi^Δ26^/TM6, Tb^1^*, 18) *y w; crb^1-7^::HA-A* (*crb^1-7-HA^*), 19) *UAS-attB-eys^wt^-VK31*, 20) *UAS-attB-eys^1-4^-VK31*, 21) *UAS-attB-eys^1-5^-VK31*, 22) *UAS-attB-rumi^wt-FLAG^-VK22*, 23) *UAS-attB-rumi^79-FLAG^-VK22* (this study), 24) *y w; crb^wt^::HA-A* (*crb^wt-HA^*) [25], 25) *crb^11A22^/TM6, Tb^1^* [10].

All *rumi* mutant crosses were raised at 18°C to minimize the temperature-dependent defects in Notch signaling unless otherwise specified. To obtain *N^55e11^/Y; PBac{N^gt-4-35^}attVK22/+* animals, *N^55e11^/FM7c* females were crossed to *y w/Y; PBac{N^gt-4-35^}attVK22* males and the male progeny were selected based on the absence of the *FM7* bar eye phenotype.

To remove one copy of *Hsc70-3* in *rumi^−/−^* animals, *w^67^ c^23^ P{lacW}Hsc70-3^G0102^/FM7c* females were first crossed to *y w; FRT82B rumi^Δ26^/TM6, Tb^1^* males. The *w^67^ c^23^ P{lacW}Hsc70-3^G0102^/y w; FRT82B rumi^Δ26^/+* female progeny from this cross were backcrossed to *y w; FRT82B rumi^Δ26^/TM6, Tb^1^* males. *w^67^ c^23^ P{lacW}Hsc70-3^G0102^/y w; FRT82B rumi^Δ26^/FRT82B rumi^Δ26^* progeny were selected based on the eye color from the *Hsc70-3* allele and the *rumi* mutant bristle phenotype [14] and used for TEM analysis.

### *O*-Glucose site mapping on *Drosophila* Crb and Eys fragments expressed in S2 cells

A construct encoding EGF1-5 from *Drosophila* Eys (harboring four out of the five Rumi target sites of Eys) was synthesized (Genewiz, Inc.). EGF12-17 from *Drosophila* Crb (harboring five out of the seven Rumi targets sites of Crb) was amplified using region-specific primers from genomic DNA extracted from flies carrying a *UAS-crb-full-length* transgene [20]. The genomic DNA was obtained using a DNA purification kit from Promega. The Eys fragment was cloned in frame to an N-terminal signal peptide from *Drosophila* Acetylcholine esterase and C-terminal V5 and 6x-Histidine tags in the *pMT/V5-HisB-ACE* vector [12]. The Crb fragment was cloned into a *pMT/BiP/3xFLAG* vector using *EcoRI* and *XbaI* [45]. Eys-EGF1-5-V5-His and Crb-EGF12-17-3xFLAG were expressed in *Drosophila* S2 cells, purified from medium by Nickel column or anti-FLAG resin, respectively, reduced and alkylated, and subjected to in-gel protease digests as described [46], [47] with minor modifications. *O*-Glucose modified glycopeptides were identified by neutral loss of the glycans during collision-induced dissociation (CID) using nano-LC-MS/MS as described [13].

### Dissections, staining, processing, image acquisition and quantification

For dissection at 55% and 65% pupal development, animals were selected at the white prepupal stage and aged for 4.5 days (55%) and 5.5 days (65%) at 18°C. For animals raised at higher temperatures, the white prepupae were placed at 25°C at zero hours APF for 1 day and subsequently placed at 30°C until 55% or 75% PD for dissection. The pupal case was removed and heads were pierced to allow proper fixation. Corneas were removed from the eyes in PBS. Tissues were fixed using 4% formaldehyde for 30–40 minutes, and then washed in 0.3–0.5% Triton X-100 in PBS. Blocking and antibody incubations were performed in PBS containing 0.5% Triton X-100 and 5% Serum (Donkey or Goat). The following antibodies were used: mouse anti-Eys (21A6) 1∶250 and mouse anti-ELAV (9F8A9) 1∶200 (Developmental Studies Hybridoma Bank), guinea pig anti-Eys 1∶1000 [5], guinea pig anti-Boca 1∶1000 [48], rat anti-Crb 1∶500 [11], rabbit anti-Lava lamp 1∶2000 [49], rabbit anti-Rab11 1∶1000 [50], Rabbit anti-Rab7 1∶100 [51], mouse anti-Rab11 1∶100 (BD Biosciences), guinea pig anti-Senseless 1∶2000 [52], goat anti-mouse-Cy3 1∶500, goat anti-mouse-Cy5 1∶500, donkey anti-mouse-Dylight649 1∶500, donkey anti-mouse-Cy3 1∶500, donkey anti-rabbit-Cy3 1∶500, donkey anti-guinea pig-Dylight649 1∶500 (Jackson ImmunoResearch Laboratories). Phalloidin Alexa488 conjugated 1∶500 (Life Technologies) was used to visualize rhabdomeres. Confocal images were taken with either a Leica TCS-SP5 microscope with an HCX-PL-APO oil 63x, NA 1.25 objective and an HCX-PL-APO 20x, 0.7 NA objective with a PMT SP confocal detector, or a TCS-SP8 microscope with an HC-PL-APO glycerol 63x, NA 1.3 objective and HyD SP GaAsp detector. All images were acquired using Leica LAS-SP software. Amira 5.2.2 and Adobe Photoshop CS4 were used for processing and figures were assembled in Adobe Illustrator CS5.1.

To quantify IRS size, the electron micrographs were opened using “Fiji is just ImageJ” open source image processing software. The scale was set by tracing the scale bar in the image using the line tool and using the “set scale” function. The IRS was traced using the freehand selection tool and the area was measured using the “measure” function.

To quantify total pixel intensity, the Amira 5.2.2 image processing software was used. A single ommatidium was cropped, which was done twice for each image to obtain data from 2 different ommatidia per animal. The desired channel for quantification was labeled with the “label field” function, and the “segmentation editor” was opened. The IRS was traced using the lasso freehand tool, placed in a separate “material”, and the rest of the pixels in the channel were selected using the threshold tool and placed in a separate “material”. The same threshold was used for all ommatidia. In the “object pool” module, the total pixel intensities for IRS and the rest of the ommatidium were obtained using the “material statistics” option.

### Western blotting

Proteins were extracted by lysing the heads in RIPA buffer (Boston BioProducts) containing a dissolved protease inhibitor cocktail tablet (Roche Diagnostics). Approximately 10 µL RIPA buffer was used per fly head. The following antibodies were used: guinea pig anti-Hsc70-3 1∶5000 [27], guinea pig anti-Eys 1∶10000 [5], mouse anti-FLAG 1∶1000 (M2, Sigma-Aldrich), mouse anti-Tubulin 1∶1000 (Santa Cruz Biotech), goat anti-guinea pig-HRP 1∶5000 and goat anti-mouse-HRP 1∶5000 (Jackson ImmunoResearch Laboratories). Western blots were developed using Pierce ECL Western Blotting Substrates (Thermo Scientific). The bands were detected and quantified using an ImageQuant LAS 4000 system and ImageQuant TL software, respectively, from GE Healthcare. At least two independent immunoblots were performed for each experiment.

### Transmission electron microscopy

To process flies using transmission electron microscopy, heads were dissected and fixed overnight at 4°C in paraformaldehyde, glutaraldehyde and cacodylic acid and processed as previously described [53]. Briefly, after fixation, heads were post fixed with 1–2% OsO_4_, dehydrated with ethanol and propylene oxide, and then embedded in Embed-812 resin. Thin sections (∼50 nm thick) were stained with 1–2% uranyl acetate as the negative stain and then stained with Reynold's lead citrate. Images were obtained using three different transmission electron microscopes: 1) Hitachi H-7500 with a Gatan US100 camera: images were captured using Digital Micrograph, v1.82.366 software; 2) JEOL 1230 with a Gatan Ultrascan 1000 camera: images were captured with Gatan Digital Micrograph software; 3) JEOL JEM 1010 with an AMT XR-16 camera: images were captured using AMT Image Capture Engine V602. All images were processed using Adobe Photoshop CS4 and figures were assembled in Adobe Illustrator CS5.1.

### Generation of the knock-in and transgenic animals

To generate the *crb^1-7^::HA-A* knock-in allele (*crb^1-7-HA^*), a *crb* mutant founder line was used in which 10 kb of the *crb* locus harboring most of the coding region is replaced with an *attP* and a *loxP* site [25]. Multiple rounds of end overlap PCR were used to introduce serine-to-alanine mutations in all seven Rumi target sites of Crb in the *pGE-attB^GMR^-crb^wt^::HA-A* targeting vector [25] to generate the *pGE-attB^GMR^-crb^1-7^::HA-A* targeting construct. ΦC31-mediated integration was used to introduce the *crb^1-7^::HA-A* fragment into the *crb* locus of the *crb* mutant founder line. A Cre-expressing transgene [54] was used to remove the *GMR-hsp::white* and the remaining vector sequences from the knock-in allele and to obtain the *white^−^* allele *crb^1-7^::HA-A* used in our study. Genomic PCR with multiple primer pairs in the region was performed to confirm correct integration and Cre-mediated recombination, as described previously [25]. Primer sequences are available upon request.

To generate the wild-type and mutant *eys* transgenes, the full length *eys* cDNA was retrieved from the *pUAST-eys* construct [6] using restriction digestion and cloned into the *pUASTattB* vector [28], resulting in *pUASTattB-eys^wt^*. To generate the mutant *eys* transgenes, a 603-bp cDNA fragment containing EGF1-5 of Eys with serine-to-alanine mutations in the four target sites in this region (EGF1-3 and EGF5) was synthesized (Genewiz, Inc.). The wild-type EGF1-5 region in *pUASTattB-eys^wt^* construct was replaced with the synthesized mutant version using two rounds of end-overlap PCR [55] with three overlapping fragments. The resulting 1.2-kb fragment containing the first four mutant Rumi target sites was placed in *pUASTattB-eys^wt^* by using *BglII* and *SacII* restriction enzymes to generate *pUASTattB-eys^1-4^*. To mutate the fifth (last) Rumi target site in *eys*, a 4-kb fragment of the *eys* cDNA containing the target site (EGF9) and flanked by *NdeI* and *KpnI* restriction sites was PCR amplified using Phusion DNA polymerase (New England Biolabs). The PCR product was cloned into *pSC-B* using the Strataclone blunt PCR cloning kit (Agilent Technologies) to generate *pSC-B-Eys-EGF9*. Site-directed mutagenesis was performed using complementary primers and Phusion DNA polymerase to introduce the serine-to-alanine mutation. The wild-type 4-kb fragment from *pUASTattB-eys^1-4^* was replaced with the mutant version by using *NdeI* and *KpnI* to generate *pUASTattB-eys^1-5^*. All three constructs were integrated into the *VK31* docking site using standard methods [28], [29]. Correct integration was confirmed by PCR.

To generate wild-type and mutant *rumi* transgenes, FLAG-tagged versions of *rumi^wt^* and *rumi^79^* (G189E) ORF were excised from *pUAST-rumi^wt-FLAG^* and *pUAST-rumi^79-FLAG^* [12] by using *EcoRI*-*KpnI* double digestion and were cloned into the *pUAST-attB* vector [28]. After verification by sequencing, the constructs were integrated into the *VK22* docking site using standard methods and verified by PCR [28], [29].

### Statistical analysis

Data are presented as mean ± SEM. To compare the number of rhabdomere clusters per ommatidium, ANOVA with Scheffé or Tukey multiple comparisons or *t*-test was performed. To compare the IRS size between wild-type and *rumi* ommatidia at 65% PD, unpaired *t*-test was used.

## Supporting Information

Figure S1 *rumi^79^* is not likely to be a dominant negative allele. (A–C′) Overexpression of FLAG-tagged versions of wild-type Rumi (A,A′) or Rumi^79^ (B,B′; G189E) by *GMR-GAL4* does not impair rhabdomere separation. Note the absence of attachments between rhabdomeres marked by Phalloidin (green) and the continuous expression of Eys (red) in animals overexpressing wild-type and mutant Rumi (A–B′), similar to the control *GMR-GAL4/+* animals (C,C′). Scale bar in A is 5 µm and applies to A–C′. (D) Western blotting with anti-FLAG antibody shows that wild-type and G189E Rumi are overexpressed at comparable levels in *GMR>rumi^wt-FLAG^* and *GMR>rumi^79-FLAG^* animals.
(TIF)

Figure S2 Rumi target sites in Crb EGF12, EGF13 and EGF15 are *O*-glucosylated. (A) Identification of the peptide ^671^CYCTPGFTGVHCDSDVDECLSFPCLNGATCHNK^703^ from Crb EGF12. Amino acid numbering for all Crb peptides is based on the Crb-PA polypeptide (FlyBase ID: FBpp0083987). The top panel shows a full MS spectrum of material eluting at 8.1 min. The ion labeled [M+4H] ^4+^ matches the predicted mass for quadruply charged form of the peptide modified with *O*-fucose monosaccharide and *O*-glucose monosaccharide. Other ions are from co-eluting material. CID fragmentation of the quadruply charged form of this peptide, m/z 1048.3 (top panel, [M+4H] ^4+^), resulted in the MS/MS spectrum shown in the bottom panel. Numerous sequence fragment ions (arrows) are observed that confirm the identity of the peptide. The position of the parent ion fragmented in the MS/MS spectrum is identified with a blue diamond. The blue underlined S is the glucosylated serine, and the red underlined T is the fucosylated threonine. (B) Identification of the peptide ^704^INAYECVCQPGYEGENCEVDIDECGSNPCSNGSTCIDR^741^ from Crb EGF13. The top panel shows a full MS spectrum of material eluting at 6.6 min. The ion labeled [M+4H] ^4+^ matches the predicted mass for quadruply charged form of the peptide modified with *O*-fucose monosaccharide and *O*-glucose monosaccharide. Other ions are from co-eluting material. CID fragmentation of the quadruply charged form of this peptide, m/z 1185.7 (top panel, [M+4H] ^4+^), resulted in the MS/MS spectrum shown in the bottom panel. Numerous sequence fragment ions (arrows) are observed that confirm the identity of the peptide. The position of the parent ion fragmented in the MS/MS spectrum is identified with a blue diamond. (C) Identification of the peptide ^806^SNPCTNGAKCL^816^ from Crb EGF15. The top panel shows a full MS spectrum of material eluting at 3.1 min. The ion labeled [M+2H]^2+^ matches the predicted mass for doubly charged form of the peptide modified with *O*-glucose monosaccharide. Other ions are from co-eluting material. CID fragmentation of the doubly charged form of this peptide, m/z 693.1 (top panel, [M+2H]^2+^), resulted in the MS/MS spectrum shown in the bottom panel. Numerous sequence fragment ions (arrows) are observed that confirm the identity of the peptide. The position of the parent ion fragmented in the MS/MS spectrum is identified with a blue diamond. In all MS/MS spectra, ions representing glycopeptides are indicated by black lines modified with *O*-glucose (blue circle) and *O*-fucose (red triangle). Ions representing the glycopeptides in the MS spectra were used to generate EIC in Figure 3B–D.
(TIF)

Figure S3 Rumi target sites in Crb EGF16 and EGF17 are *O*-glucosylated. (A) Identification of the peptide ^831^KGKNCEQDINECESNPCQY^849^ from Crb EGF16. The top panel shows a full MS spectrum of material eluting at 4 min. The ion labeled [M+3H]^3+^ matches the predicted mass for triply charged form of the peptide modified with *O*-glucose monosaccharide. Other ions are from co-eluting material. CID fragmentation of the triply charged form of this peptide, m/z 846.2 (top panel, [M+3H]^3+^), resulted in the MS/MS spectrum shown in the bottom panel. Numerous sequence fragment ions (arrows) are observed that confirm the identity of the peptide. Ions selected for fragmentation in the MS spectrum are identified by red diamonds. The position of the parent ion fragmented in the MS/MS spectrum is identified with a blue diamond. The blue underlined S is the glucosylated serine. (B) Identification of the peptide ^898^NCEININECDSNPCSK^913^ from Crb EGF17. The top panel shows a full MS spectrum of material eluting at 4.6 min. The ion labeled [M+3H]^3+^ matches the predicted mass for triply charged form of the peptide modified with *O*-glucose monosaccharide, and the ion labeled [M+2H]2^+^ matches the predicted mass for doubly charged form of the same peptide. Other ions are from co-eluting material. CID fragmentation of the doubly charged form of this peptide, m/z 1059.1 (top panel, [M+2H]^2+^), resulted in the MS/MS spectrum shown in the middle panel. CID fragmentation of the triply charged form of this peptide, m/z 706.4 (top panel, [M+3H]^3+^), resulted in the MS/MS spectrum shown in the bottom panel. Numerous sequence fragment ions (arrows) are observed that confirm the identity of the peptide. Ions selected for fragmentation in the MS spectrum are identified by red diamonds. The positions of the parent ions fragmented in the MS/MS spectrum are identified with blue diamonds. In all MS/MS spectra, ions representing glycopeptides are indicated by black lines modified with *O*-glucose (blue circle). Ions representing the glycopeptides in the MS spectra were used to generate EIC in Figure 3E and 3F.
(TIF)

Figure S4 Rumi target sites in Eys EGF1, EGF2 and EGF5 are *O*-glucosylated. (A–C) Top: full MS spectra of Eys EGF1 (A), EGF2 (B) and EGF5 (C) peptides. Bottom: CID fragmentation of the doubly charged (A and B) or triply charged (C) forms of the identified peptide. The blue underlined S is the glucosylated serine, and the red underlined T is the fucosylated threonine. (A) Identification of the peptide ^147^ACLSNPCVF^155^ from Eys EGF1. Amino acid numbering for all Eys peptides is based on Eys-PE polypeptide (FlyBase ID: FBpp0311004). The top panel shows a full MS spectrum of material eluting at 6.3 min. The ions labeled [M+2H]^2+^ match the predicted mass for doubly charged forms of the unmodified peptide (m/z 534.3) and the peptide modified with an *O*-glucose and a xylose (m/z 680.8). Other ions are from co-eluting material. CID fragmentation of the doubly charged form of the unmodified peptide, m/z 534.3 (top panel, [M+2H]^2+^ to the left), resulted in the MS/MS spectrum shown in the middle panel. CID fragmentation of the doubly charged form of the glycosylated peptide, m/z 680.8 (top panel, [M+2H]^2+^ to the right), resulted in the MS/MS spectrum shown in the bottom panel. Numerous sequence fragment ions (arrows) are observed that confirm the identity of the peptides. (B) Identification of the peptide ^188^SSPCQNGGTCVDGVAYY^204^ from Eys EGF2. The top panel shows a full MS spectrum of material eluting at 6.8 min. The ion labeled [M+2H]^2+^ matches the predicted mass for doubly charged form of the peptide modified with *O*-glucose monosaccharide and *O*-fucose monosaccharide. Other ions are from co-eluting material. CID fragmentation of the doubly charged form of this peptide, m/z 1073.3 (top panel, [M+2H]^2+^), resulted in the MS/MS spectrum shown in the bottom panel. Numerous sequence fragment ions (arrows) are observed that confirm the identity of the peptide. (C) Identification of the peptide ^282^HGRICQEEINECASSPCQNGGVCVDKLAAY^322^ from Eys EGF5. The top panel shows a full MS spectrum of material eluting at 7.2 min. The ion labeled [M+3H]^3+^ matches the predicted mass for triply charged form of the peptide modified with *O*-glucose monosaccharide. Other ions are from co-eluting material. CID fragmentation of the triply charged form of this peptide, m/z 1196.7 (top panel, [M+3H]^3+^), resulted in the MS/MS spectrum shown in the bottom panel. Numerous sequence fragment ions (arrows) are observed that confirm the identity of the peptide. Ions selected for fragmentation in the MS spectrum are identified by red diamonds. The position of the parent ion fragmented in the MS/MS spectrum is identified with a blue diamond. In all MS/MS spectra, ions representing glycopeptides are indicated by black lines modified with *O*-glucose (blue circle) and *O*-fucose (red triangle). Ions representing the glycopeptides in the MS spectra shown here were used to generate the EICs in Figure 4B, 4C and 4D.
(TIF)
