# Supplementary figures and images for: The Protein O-glucosyltransferase Rumi Modifies Eyes Shut to Promote Rhabdomere Separation in Drosophila
Source: PLoS Genet. 2014 Nov 20;10(11):e1004795. doi: 10.1371/journal.pgen.1004795 (PMC4238978; doi:10.1371/journal.pgen.1004795)

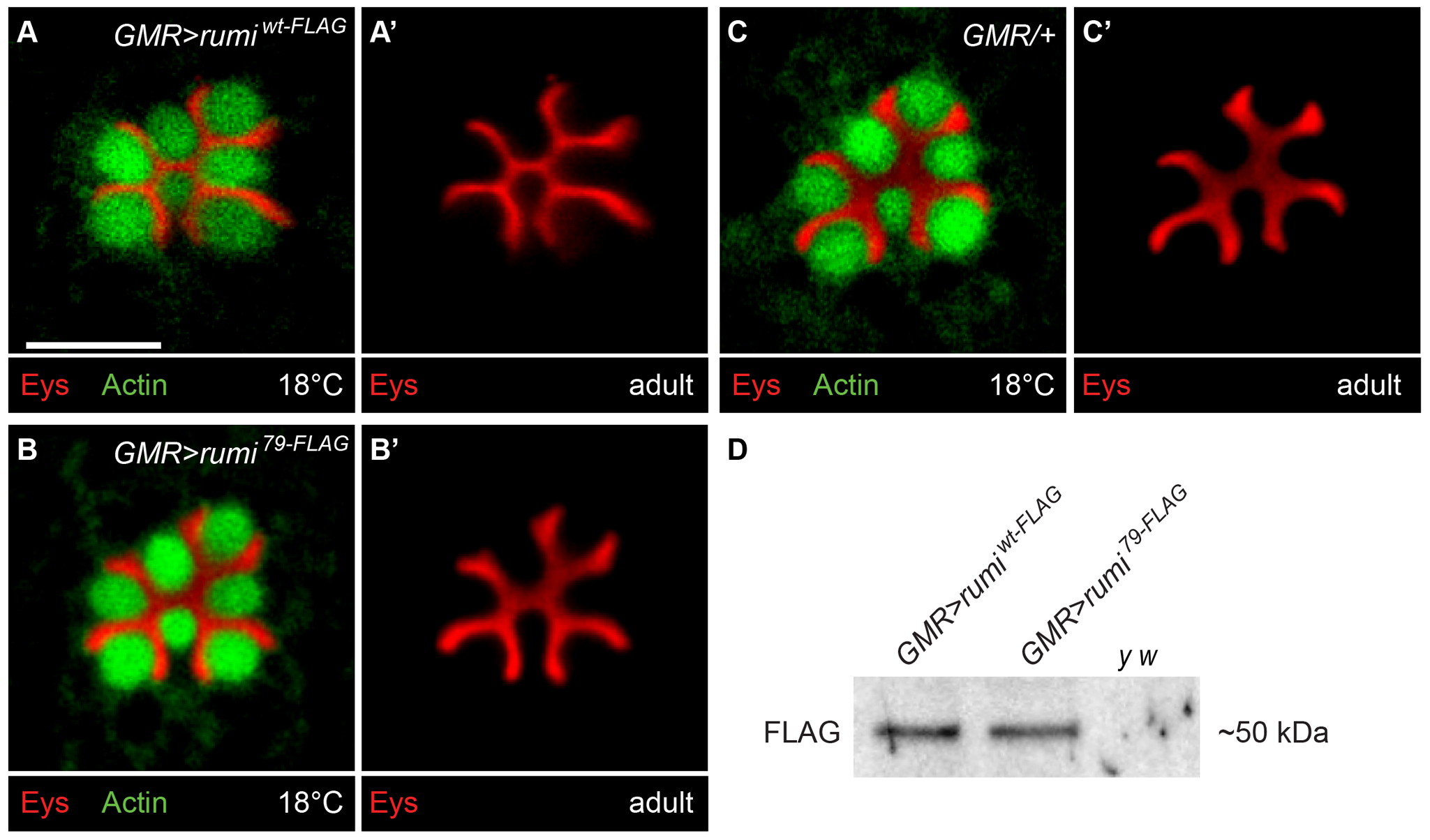

Supplement: Figure S1 — rumi79 is not likely to be a dominant negative allele. (A–C′) Overexpression of FLAG-tagged versions of wild-type Rumi (A,A′) or Rumi79 (B,B′; G189E) by GMR-GAL4 does not impair rhabdomere separation. Note the absence of attachments between rhabdomeres marked by Phalloidin (green) and the continuous expression of Eys (red) in animals overexpressing wild-type and mutant Rumi (A–B′), similar to the control GMR-GAL4/+ animals (C,C′). Scale bar in A is 5 µm and applies to A–C′. (D) Western blotting with anti-FLAG antibody shows that wild-type and G189E Rumi are overexpressed at comparable levels in GMR>rumiwt-FLAG and GMR>rumi79-FLAG animals. (TIF) [file pgen.1004795.s001.tif]

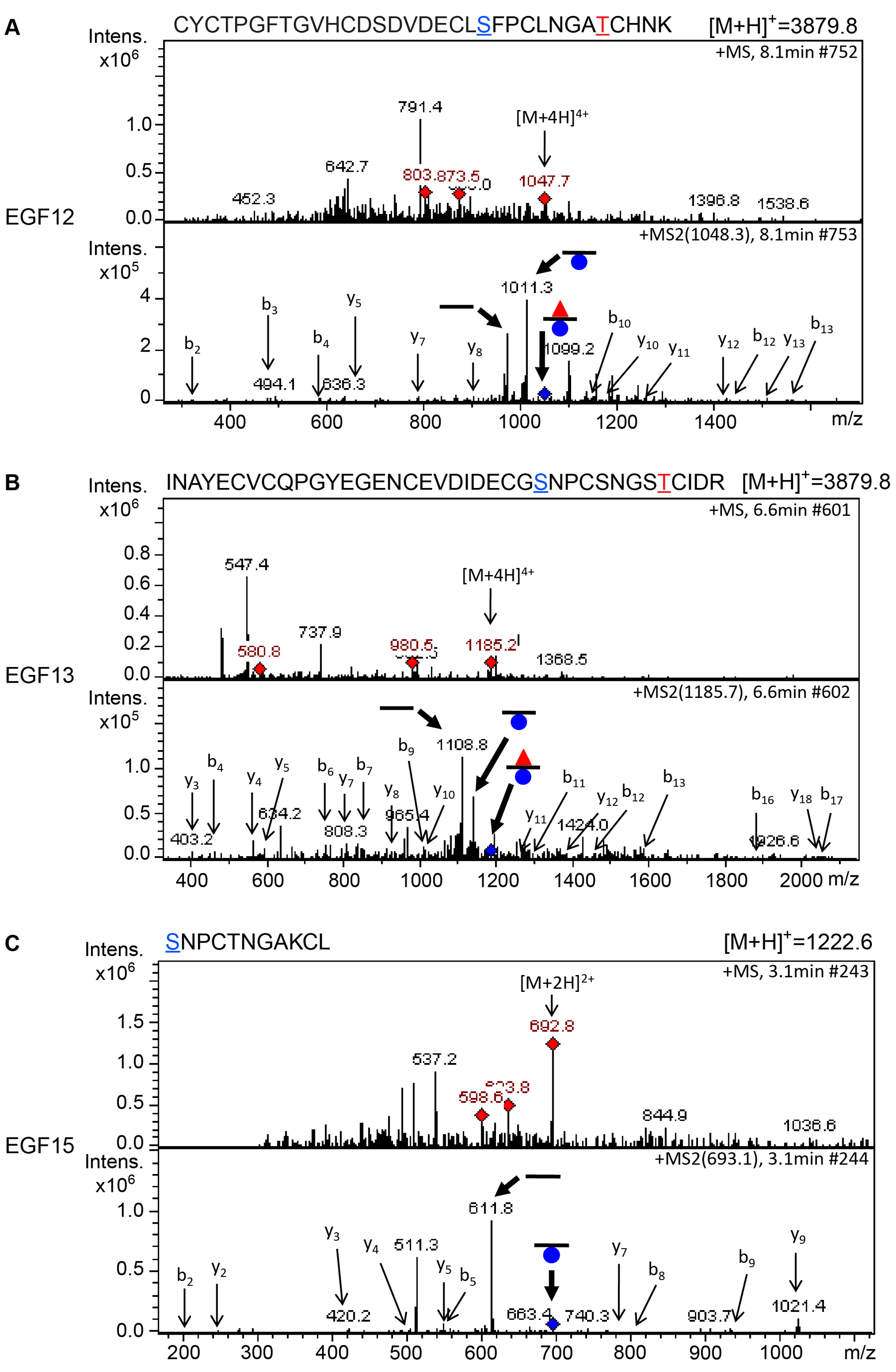

Supplement: Figure S2 — Rumi target sites in Crb EGF12, EGF13 and EGF15 are O-glucosylated. (A) Identification of the peptide 671CYCTPGFTGVHCDSDVDECLSFPCLNGATCHNK703 from Crb EGF12. Amino acid numbering for all Crb peptides is based on the Crb-PA polypeptide (FlyBase ID: FBpp0083987). The top panel shows a full MS spectrum of material eluting at 8.1 min. The ion labeled [M+4H] 4+ matches the predicted mass for quadruply charged form of the peptide modified with O-fucose monosaccharide and O-glucose monosaccharide. Other ions are from co-eluting material. CID fragmentation of the quadruply charged form of this peptide, m/z 1048.3 (top panel, [M+4H] 4+), resulted in the MS/MS spectrum shown in the bottom panel. Numerous sequence fragment ions (arrows) are observed that confirm the identity of the peptide. The position of the parent ion fragmented in the MS/MS spectrum is identified with a blue diamond. The blue underlined S is the glucosylated serine, and the red underlined T is the fucosylated threonine. (B) Identification of the peptide 704INAYECVCQPGYEGENCEVDIDECGSNPCSNGSTCIDR741 from Crb EGF13. The top panel shows a full MS spectrum of material eluting at 6.6 min. The ion labeled [M+4H] 4+ matches the predicted mass for quadruply charged form of the peptide modified with O-fucose monosaccharide and O-glucose monosaccharide. Other ions are from co-eluting material. CID fragmentation of the quadruply charged form of this peptide, m/z 1185.7 (top panel, [M+4H] 4+), resulted in the MS/MS spectrum shown in the bottom panel. Numerous sequence fragment ions (arrows) are observed that confirm the identity of the peptide. The position of the parent ion fragmented in the MS/MS spectrum is identified with a blue diamond. (C) Identification of the peptide 806SNPCTNGAKCL816 from Crb EGF15. The top panel shows a full MS spectrum of material eluting at 3.1 min. The ion labeled [M+2H]2+ matches the predicted mass for doubly charged form of the peptide modified with O-glucose monosaccharide. Other ions a [file pgen.1004795.s002.tif]

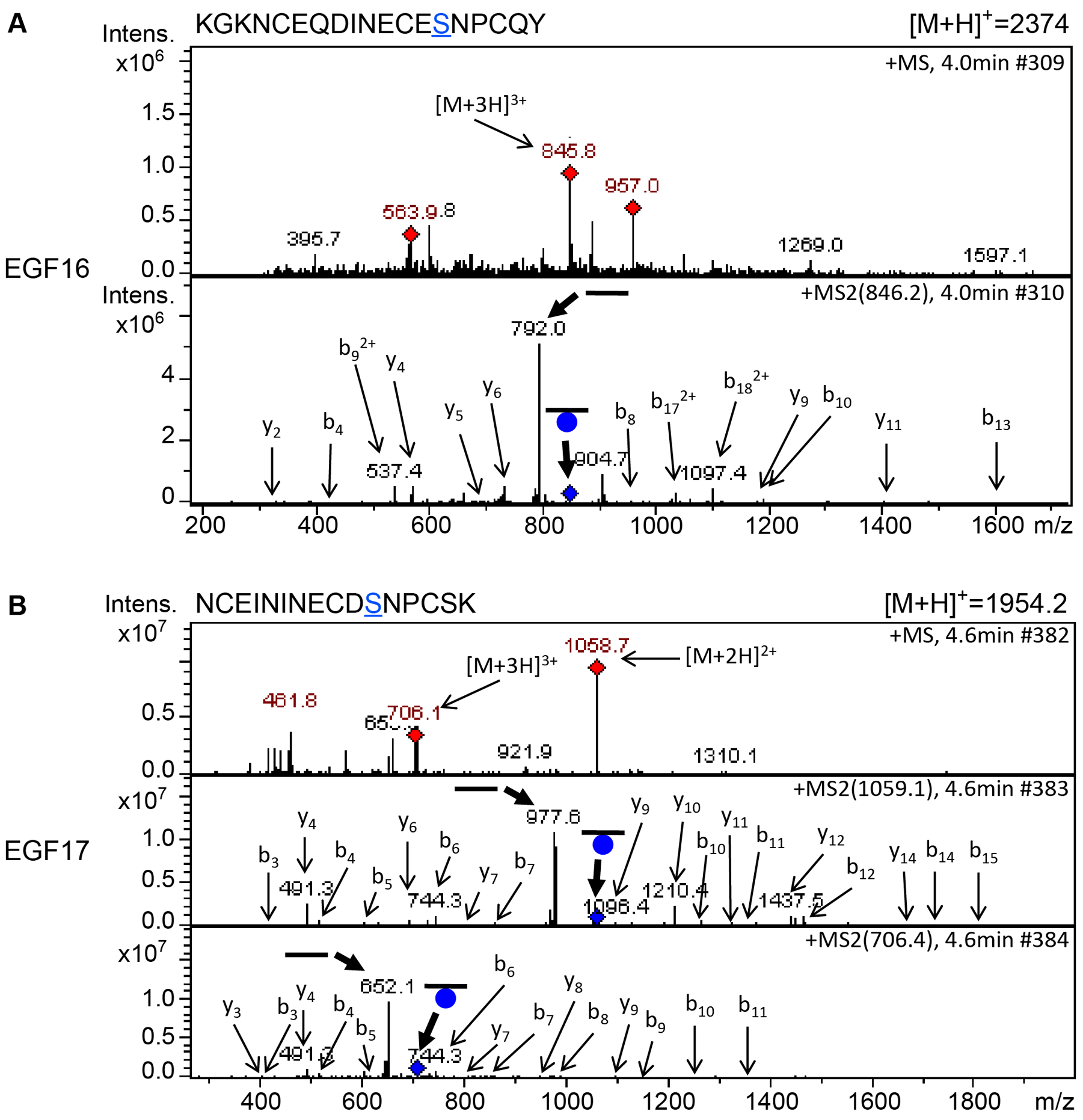

Supplement: Figure S3 — Rumi target sites in Crb EGF16 and EGF17 are O-glucosylated. (A) Identification of the peptide 831KGKNCEQDINECESNPCQY849 from Crb EGF16. The top panel shows a full MS spectrum of material eluting at 4 min. The ion labeled [M+3H]3+ matches the predicted mass for triply charged form of the peptide modified with O-glucose monosaccharide. Other ions are from co-eluting material. CID fragmentation of the triply charged form of this peptide, m/z 846.2 (top panel, [M+3H]3+), resulted in the MS/MS spectrum shown in the bottom panel. Numerous sequence fragment ions (arrows) are observed that confirm the identity of the peptide. Ions selected for fragmentation in the MS spectrum are identified by red diamonds. The position of the parent ion fragmented in the MS/MS spectrum is identified with a blue diamond. The blue underlined S is the glucosylated serine. (B) Identification of the peptide 898NCEININECDSNPCSK913 from Crb EGF17. The top panel shows a full MS spectrum of material eluting at 4.6 min. The ion labeled [M+3H]3+ matches the predicted mass for triply charged form of the peptide modified with O-glucose monosaccharide, and the ion labeled [M+2H]2+ matches the predicted mass for doubly charged form of the same peptide. Other ions are from co-eluting material. CID fragmentation of the doubly charged form of this peptide, m/z 1059.1 (top panel, [M+2H]2+), resulted in the MS/MS spectrum shown in the middle panel. CID fragmentation of the triply charged form of this peptide, m/z 706.4 (top panel, [M+3H]3+), resulted in the MS/MS spectrum shown in the bottom panel. Numerous sequence fragment ions (arrows) are observed that confirm the identity of the peptide. Ions selected for fragmentation in the MS spectrum are identified by red diamonds. The positions of the parent ions fragmented in the MS/MS spectrum are identified with blue diamonds. In all MS/MS spectra, ions representing glycopeptides are indicated by black lines modified with O-glucose (blue circle). Ions representi [file pgen.1004795.s003.tif]

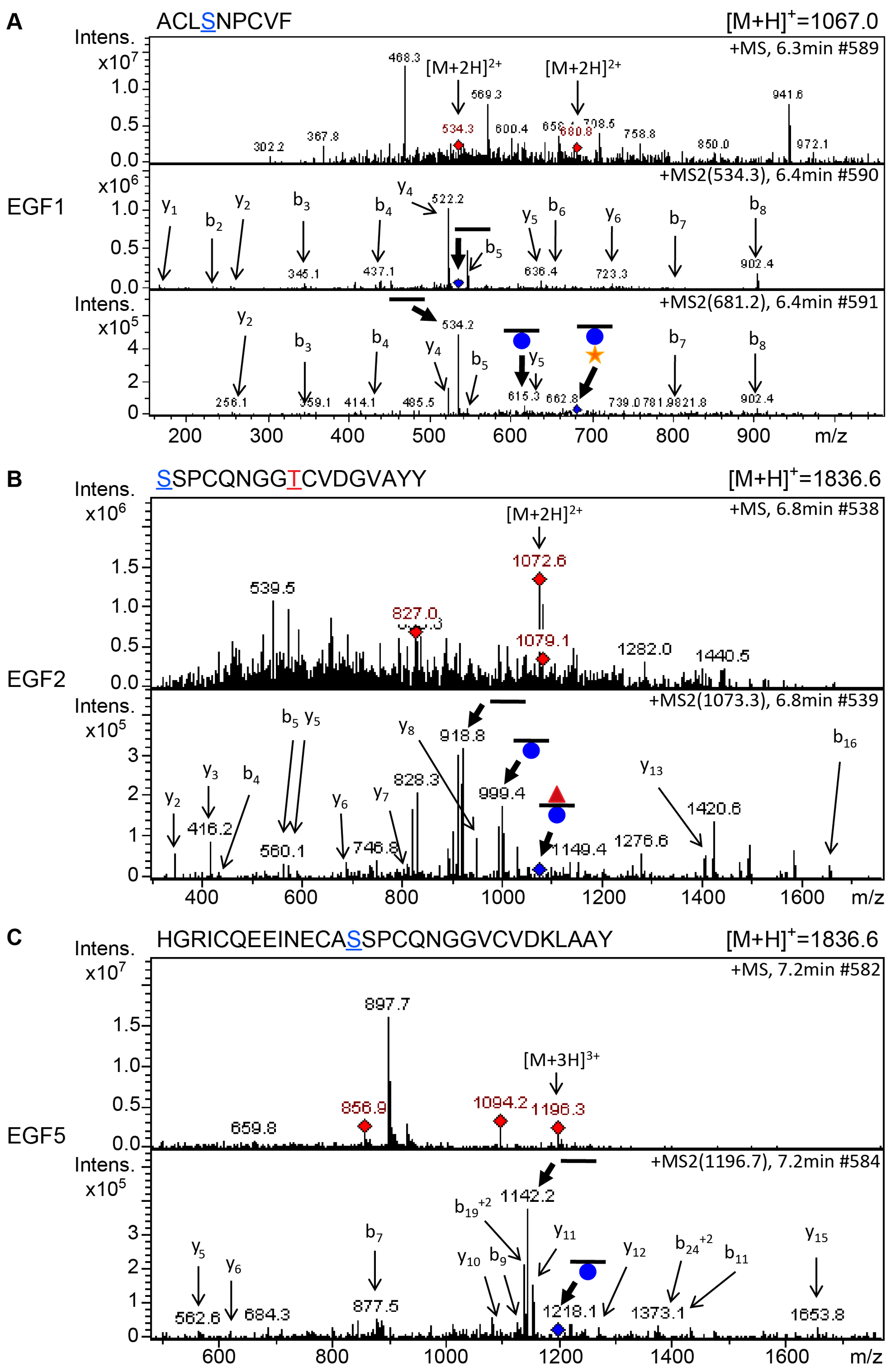

Supplement: Figure S4 — Rumi target sites in Eys EGF1, EGF2 and EGF5 are O-glucosylated. (A–C) Top: full MS spectra of Eys EGF1 (A), EGF2 (B) and EGF5 (C) peptides. Bottom: CID fragmentation of the doubly charged (A and B) or triply charged (C) forms of the identified peptide. The blue underlined S is the glucosylated serine, and the red underlined T is the fucosylated threonine. (A) Identification of the peptide 147ACLSNPCVF155 from Eys EGF1. Amino acid numbering for all Eys peptides is based on Eys-PE polypeptide (FlyBase ID: FBpp0311004). The top panel shows a full MS spectrum of material eluting at 6.3 min. The ions labeled [M+2H]2+ match the predicted mass for doubly charged forms of the unmodified peptide (m/z 534.3) and the peptide modified with an O-glucose and a xylose (m/z 680.8). Other ions are from co-eluting material. CID fragmentation of the doubly charged form of the unmodified peptide, m/z 534.3 (top panel, [M+2H]2+ to the left), resulted in the MS/MS spectrum shown in the middle panel. CID fragmentation of the doubly charged form of the glycosylated peptide, m/z 680.8 (top panel, [M+2H]2+ to the right), resulted in the MS/MS spectrum shown in the bottom panel. Numerous sequence fragment ions (arrows) are observed that confirm the identity of the peptides. (B) Identification of the peptide 188SSPCQNGGTCVDGVAYY204 from Eys EGF2. The top panel shows a full MS spectrum of material eluting at 6.8 min. The ion labeled [M+2H]2+ matches the predicted mass for doubly charged form of the peptide modified with O-glucose monosaccharide and O-fucose monosaccharide. Other ions are from co-eluting material. CID fragmentation of the doubly charged form of this peptide, m/z 1073.3 (top panel, [M+2H]2+), resulted in the MS/MS spectrum shown in the bottom panel. Numerous sequence fragment ions (arrows) are observed that confirm the identity of the peptide. (C) Identification of the peptide 282HGRICQEEINECASSPCQNGGVCVDKLAAY322 from Eys EGF5. The top panel shows a full MS spectrum of material [file pgen.1004795.s004.tif]
